# Subnational Projections of Lymphatic Filariasis Elimination Targets in Ethiopia to Support National Level Policy

**DOI:** 10.1093/cid/ciae072

**Published:** 2024-04-25

**Authors:** Joaquin M Prada, Panayiota Touloupou, Biruck Kebede, Emanuelle Giorgi, Heven Sime, Morgan Smith, Periklis Kontoroupis, Paul Brown, Jorge Cano, Hajnal Farkas, Mike Irvine, Lisa Reimer, Rocio Caja Rivera, Sake J de Vlas, Edwin Michael, Wilma A Stolk, Rachel Pulan, Simon E F Spencer, T Déirdre Hollingsworth, Fikre Seife

**Affiliations:** Department of Comparative Biomedical Sciences, Faculty of Health and Medical Sciences, University of Surrey, Guildford, United Kingdom; School of Mathematics, University of Birmingham, Birmingham, United Kingdom; RTI International, 3040 E Cornwallis Rd, Research Triangle Park, North Carolina 27709, USA; CHICAS, Lancaster University, Lancaster, United Kingdom; Malaria and Neglected Tropical Diseases Research Team, Bacterial, Parasitic and Zoonotic Disease Research Directorate, Ethiopian Public Health Institute, Addis Ababa, Ethiopia; Department of Biological Sciences, University of Notre Dame, Notre Dame, Indiana, USA; Erasmus MC, University Medical Center Rotterdam, Rotterdam, The Netherlands; Zeeman Institute for Systems Biology & Infectious Disease Epidemiology Research, University of Warwick, Coventry, United Kingdom; Expanded Special Project for Elimination of Neglected Tropical Diseases (ESPEN), WHO Regional Office for Africa, Brazzaville, Democratic Republic of the Congo; Zeeman Institute for Systems Biology & Infectious Disease Epidemiology Research, University of Warwick, Coventry, United Kingdom; Faculty of Science, BC Centre for Disease Control, Vancouver, Canada; Department of Vector Biology, Liverpool School of Tropical Medicine, Liverpool, United Kingdom; College of Public Health, University of South Florida, Tampa, Florida, USA; Erasmus MC, University Medical Center Rotterdam, Rotterdam, The Netherlands; College of Public Health, University of South Florida, Tampa, Florida, USA; Erasmus MC, University Medical Center Rotterdam, Rotterdam, The Netherlands; Faculty of Infectious and Tropical Diseases, London School of Hygiene & Tropical Medicine, London, United Kingdom; Zeeman Institute for Systems Biology & Infectious Disease Epidemiology Research, University of Warwick, Coventry, United Kingdom; Nuffield Department of Medicine, Big Data Institute, Li Ka Shing Centre for Health Information and Discovery, University of Oxford, Oxford, United Kingdom; Disease Prevention and Control Directorate, Federal Ministry of Health, Addis Ababa, Ethiopia

**Keywords:** lymphatic filariasis, model, Ethiopia, geospatial, projections

## Abstract

**Background:**

Lymphatic filariasis (LF) is a debilitating, poverty-promoting, neglected tropical disease (NTD) targeted for worldwide elimination as a public health problem (EPHP) by 2030. Evaluating progress towards this target for national programmes is challenging, due to differences in disease transmission and interventions at the subnational level. Mathematical models can help address these challenges by capturing spatial heterogeneities and evaluating progress towards LF elimination and how different interventions could be leveraged to achieve elimination by 2030.

**Methods:**

Here we used a novel approach to combine historical geo-spatial disease prevalence maps of LF in Ethiopia with 3 contemporary disease transmission models to project trends in infection under different intervention scenarios at subnational level.

**Results:**

Our findings show that local context, particularly the coverage of interventions, is an important determinant for the success of control and elimination programmes. Furthermore, although current strategies seem sufficient to achieve LF elimination by 2030, some areas may benefit from the implementation of alternative strategies, such as using enhanced coverage or increased frequency, to accelerate progress towards the 2030 targets.

**Conclusions:**

The combination of geospatial disease prevalence maps of LF with transmission models and intervention histories enables the projection of trends in infection at the subnational level under different control scenarios in Ethiopia. This approach, which adapts transmission models to local settings, may be useful to inform the design of optimal interventions at the subnational level in other LF endemic regions.

## Teaser

A novel approach that combines a historical geospatial disease prevalence map of lymphatic filariasis in Ethiopia with 3 contemporary disease transmission models and intervention histories enables the projection of trends in infection at the subnational level under different intervention scenarios.

Neglected tropical diseases (NTDs) are a large burden to societies worldwide, and thus global efforts are underway for their control and elimination. Among those, lymphatic filariasis (LF) is a disease with more than a billion people at risk globally, with more than 880 million individuals living in areas that require preventive chemotherapy as of 2021 [1]. LF is a mosquito-borne disease caused by filarial parasitic worms (*Wuchereria bancrofti*, *Brugia malayi*, and *Brugia timori*), with the characteristic morbidity being lymphedema (elephantiasis) and hydrocele, associated physical disability and social stigma [2].

The Global Programme to Eliminate Lymphatic Filariasis (GPELF) was established in the year 2000 to stop LF transmission by mass drug administration (MDA) of anthelminthics and to introduce morbidity management and disability prevention measures that alleviate the suffering of those affected by the disease [3]. More recently, the roadmap for NTDs 2021–2030 [4] set a target for LF of elimination as a public health problem (EPHP) by 2030, defined as a reduction in measurable prevalence of infection in endemic areas below a target threshold: 1% microfilaria (mf) or 2% antigenaemia in populations above 5 years [4]. After reaching the elimination threshold in selected sentinel sites (pre-Transmission Assessment Survey [pre-TAS]), countries can proceed with the Transmission Assessment Survey (TAS), which needs to be passed in all endemic areas to validate country-wide elimination [5].

Implementation of MDA programmes to control LF transmission has been mostly successful worldwide. Over the past 2 decades, from the 72 countries originally requiring MDA, 17 have already achieved EPHP, and 7 are under post-MDA surveillance. Out of the remaining 48 countries considered to require MDA, only 3 had not started by 2020 [1]. However, even in countries with well-established programs, some areas remain that have struggled to achieve these targets despite several rounds of treatment. This is likely due to a combination of epidemiological and operational challenges. Given the ambitious roadmap for 2030, evaluating progress with current strategies and exploring potential alternatives that can help to accelerate progress in specific areas has become increasingly relevant and has garnered a large amount of interest [6].

Mathematical models have been extensively used to assess the comparative effectiveness of alternative strategies toward set targets in many diseases, including LF [7, 8]. For example, geospatial statistical models can provide estimates of risk or disease prevalence at very fine spatial scales [9, 10] to help prioritize resources geographically. Similarly, epidemiological transmission models can project the effects of a wide array of alternative interventions and support optimisation of control strategies (see, eg, [11]). There are 3 mathematical models among those actively supporting policy making for LF elimination programmes: EPIFIL [12–15], LYMFASIM [16–18], and TRANSFIL [19, 20], which have been used in the past both independently and as an ensemble [21]. Furthermore, these epidemiological transmission models have been combined with geospatial models to provide a flexible framework able to appropriately inform policy at the relevant spatial scales [22].

Here we apply a similar approach to Ethiopia, to explore the utility of adapting these models to local settings and their ability to inform the design of optimal control and elimination programmes at the subnational level. Ethiopia has a large-scale integrated NTD program that delivers interventions to over 70 million people every year, and national mapping of LF status was completed in 2013, identifying 6.4 million people at risk of infection across 70 “woredas” (third-level administrative boundaries) endemic for LF (since then, woredas have been split into new administrative units, with these 70 areas now corresponding to 88 woredas) [23]. Ethiopia's LF elimination program started in 2009 in 5 districts (integrated with the onchocerciasis programme) and expanded gradually, with geographical coverage of MDA achieving 100% of endemic areas in 2016. Of the 88 woredas originally endemic for LF, 13 halted MDA in recent years, after more than 5 years of successful MDAs and successfully achieving EPHP targets. In 2017 and 2018, an additional 31 woredas (out of 51 in which TAS was carried out) passed the first TAS and progress continues [23–25]. However, these data highlight the heterogeneity of LF control across woredas and the need to incorporate such variation to further improve policy decisions and achieve country-wide elimination.

To investigate these issues, here we combine geostatistical mapping with transmission modelling to explore progress toward LF elimination targets in Ethiopia at the subnational level across different timeframes. In 2016, when the collaboration underlying this analysis first began (involving the NTD Modeling Consortium, Ethiopia's Ministry of Health, and the Ethiopian Public Health Institute), the critical question to address was whether the treatment strategies being used at the time would be sufficient to achieve the targets set out in the 2012–2020 World Health Organization (WHO) NTD roadmap, which included a goal to halt MDA by 2020 [26]. Since then, following limited interruptions to MDAs during the coronavirus disease 2019 (COVID-19) pandemic and the introduction of a new road map for NTDs 2021–2030 [4], Ethiopia is currently delivering treatments in LF endemic woredas with the aim of achieving EPHP by 2025 [23]. Therefore, our analyses also include post-2020 projections under different scenarios aimed at supporting decisions on whether to continue existing interventions or to expand the programme considering the 2030 roadmap, for example, by increasing MDA coverage or frequency. Collectively, these data and the extended timeframe of this project provided the unique opportunity to explore how local context influences progression towards LF elimination in Ethiopia, evaluate how subnational level predictions align with the 2020 LF prevalence data, and explore how these models can inform local authorities on areas where alternative strategies could be put in place to accelerate progress towards the 2030 targets.

## METHODS

### Study Area, History of Control and Prevalence Data

**Figure 1.**
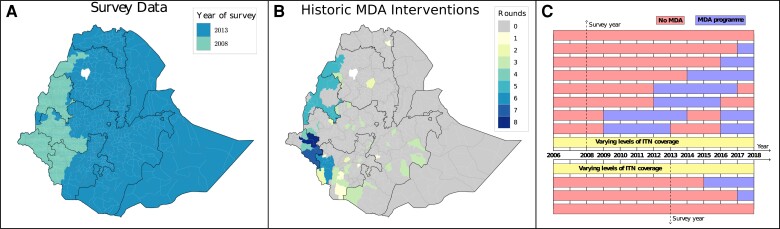
*A*, *B*, Historical data. *A* year of the survey mapping efforts (either 2008 or 2013) for each woreda; this was estimated for woredas not mapped based on their neighbors. *B,* Historic number of MDA rounds (up to 2017). *C*, Schematic of the history of control for the group of woredas in the Southwest. Woreda (n = 11) are divided into groups based on the mapping survey year (2008, top vs 2013, bottom). Each woreda has a given ITN coverage (see Supplementary Materials for details) and MDA history - years with no MDA versus years with successful MDA (≥65%). Abbreviations: ITN, insecticide treated bed-net; MDA, mass drug administration.

The 2 markers to assess the prevalence of *W. bancrofti*, the causative agent of LF in Ethiopia, are microfilaria (mf) and antigenaemia. Mf can be found in the blood of infected individuals and is also an indicator of ongoing transmission between humans and mosquitoes, with the latter potentially ingesting mf during a blood meal from an infected host. Mf counts have some disadvantages; they require night sampling (due to nocturnal periodicity in the host) and specialized parasitological skills. Antigenaemia can be measured with a simpler antigenic immunochromatographic card test (ICT), which measures adult worm antigen. However, there are challenges in its use to assess changes in transmission due to nonlinearities with mf and the effect of MDA [27]. The Filarial Test Strip (FTS) has now replaced ICT as the WHO-recommended diagnostic for mapping, monitoring, and evaluation [28]. In Ethiopia, before FTS became the recommended diagnostic tool, 2 large ICT prevalence surveys were conducted (in 2008 and 2013) for the purpose of disease mapping, Figure 1*A* .

LF is mainly focussed in 2 regions in the North-West and South-West of the country, where a few implementation units (IUs) have required MDA. In Ethiopia the woreda is the IU, which is the third administrative subdivision below regions (n = 9) and zones (n = 68). Out of the nearly 700 woredas across Ethiopia, 88 were deemed endemic for LF following the 2013 mapping, this number has since changed due to woredas split in new administrative units (96 Endemic, 34 of those under post-MDA surveillance), as of 2022 [23]. The endemic areas in Ethiopia have received multiple rounds of annual MDA with a combination of Ivermectin and Albendazole (IA), Figure 1*B* and 1*C* , starting at different times in different woredas and aiming to achieve at least 65% coverage of the total population per round [29]. The LF elimination programme has also benefited since 2006 from varying levels of insecticide treated bed-net (ITN) coverage from the malaria control programme, which has likely helped decrease transmission [30].

### Geostatistical Analysis

We generated a 5 × 5 km scale pixel map of LF ICT prevalence in Ethiopia prior to interventions using a model-based geostatistical approach (detailed in the Supplementary Material). The ICT prevalence was subsequently converted to mf prevalence following the model by Irvine et al [27], as the transmission models have been calibrated to mf prevalence (see Supplementary Material). The diagnostic tests are used only in individuals over 5 years of age, and thus the resulting map represents the mf prevalence in this sub-population, with each pixel linked to the corresponding Worldpop population estimate [31]. This map represents the infection status at the timepoint of the mapping survey, which we take as the pre-MDA baseline, Figure 1*A* . Areas in the baseline map that the geostatistical model estimated to have already achieved the target prevalence for EPHP (<1% mf) were assumed to be non-endemic and were not considered further.

### Transmission Models

We used 3 well-described published mathematical models for LF transmission and control in our analysis, developed and applied by members of the NTD Modeling Consortium. We included the following models: EPIFIL [12–15], a deterministic population-based model; and LYMFASIM [16–18] and TRANSFIL [19, 20], both stochastic individual-based models. In order to integrate these transmission models and apply them to the different pixels from the geospatial map, we first defined the parameters assumed to be fixed across our study area (Ethiopia), including those describing biological processes such as mortalities of larval stages. Then we defined the parameters that may vary spatially, such as vector density and aggregation. For these variable parameters, we defined broad prior distributions based on recent applications of these models [6, 11]. Because these models have been calibrated in the past to various settings, the fixed parameters are not discussed further. Full model and parameters descriptions are available in the Supplementary Material.

### Fitting the Transmission Models Sub-Nationally

We fitted the transmission models to the geospatial maps to provide sub-national projections following the method described below. To reduce the number of modelled scenarios, we considered that ITN coverage can be broadly divided in 4 different groups; 1 group for each of the 2 endemic regions (Northwest and Southwest of the country), which have different ITN coverage levels; another group for the remaining areas that have some ITN coverage; and the last group containing all the regions with no ITN coverage, Supplementary Figures 10–13 in the Supplementary Material. For each group we considered the average ITN coverage value per year and the most recent reported value (2015) was assumed to be maintained in future years. ITN coverage data were extracted from the Malaria Atlas Project [30] and are summarized in Supplementary Figures 10–13 in the Supplementary Material.

Within each of the 4 ITN coverage groups, areas have different survey years and MDA treatment history. For feasibility of the fitting and forward simulations, we made a simplifying assumption: if the reported MDA coverage year-by-year led to a true (achieved) MDA coverage of 65% or above, it was given the value of 65%; otherwise it was considered that no MDA had taken place. Based on this assumption, we had to consider a total of 26 alternative MDA histories. An illustrative schematic is shown in Figure 1*C* for the group of endemic woredas in the Southwest of Ethiopia.

To fit the model to the geostatistical data, we generated 100 000 simulations with each transmission model for each of the 26 treatment histories, which encompass the range of pre-control LF prevalences (ie, before MDA), population sizes, and intervention histories observed across the map. To prevent the pre-control stationary distribution from being degenerate and reduce the number of simulations being absorbed with zero prevalence, we adapted the stochastic models (LYMFASIM and TRANSFIL) to include a small rate of importation. The importation values are low enough so that in endemic areas within-pixel transmission is the dominant infection route. Once the MDA begins, disease transmission is expected to decline, so we thus progressively reduce the importation rate proportional to declines in prevalence observed in pilot simulations, Supplementary Figure 7 in the Supplementary Material.

In order to provide fine-scale predictions of prevalence after the implementation of different intervention strategies while accounting for multiple sources of spatial uncertainty, we used the Bayesian importance sampling methodology developed by Touloupou et al [22]. Each pixel belongs to 1 of the 26 treatment histories, and we selected the model simulations matching that history of MDA and ITN coverage. Those simulations are then weighted according to how closely they match the characteristics of that pixel at baseline (population size and prevalence) while accounting for the uncertainty captured in the geostatistical map. This prevents unnecessary replication of simulations for pixels that are broadly similar. The weighted ensemble of simulations from the transmission models matches the distribution of likely population size and mf prevalence in each pixel.

### Extending Projections After 2020

Given the original 2020 goals, a decision could have been made in 2020 to continue the current intervention strategy or expand the programme considering the 2030 roadmap, for example, by increasing the coverage achieved from 65% to 80%, or increasing MDA frequency from annual (aMDA) to biannual (bMDA). We considered 4 different strategies post-2020 summarized in Table 1. Each of the 26 different treatment histories can follow either of the 4 strategies post-2020, which leads to a total of 104 different scenarios that need to be considered, Supplementary Figures 10–13 in the Supplementary Material.

**Table 1.** Description of Post-2020 Strategies

Post 2020 Strategy	Description
Continue current	Finish the 5 rounds of annual MDA with 65% coverage, if already started
aMDA 65%	Continue/start/restart annual MDA (aMDA) with 65% coverage for 10 y
aMDA 80%	Switch/start aMDA with 80% coverage for 10 y
bMDA 65%	Switch/start biannual MDA (bMDA) with 65% coverage for 10 y

Abbreviation: MDA, mass drug administration.

For each pixel, we used the weights from the importance sampling to determine the projected distribution (accounting for uncertainty) in the mf prevalence under each future intervention strategy. From this distribution we calculated epidemiological outcomes such as the probability of reaching LF EPHP by 2020 and the timelines for achieving it post-2020.

The interventions are deployed at the IU level; therefore, we aggregated the pixel level results to woreda level. We took the 90th quantile in the prevalence distribution of each pixel in a woreda, weighted by population size, which represents the IU-level prevalence. If this weighted prevalence is below 1%, this represents a 90% probability of achieving elimination. As we take the same quantile for all the pixels, this implicitly assumes that all pixels within the same woreda are correlated (full Spearman rank correlation). We additionally explored 2 different certainty levels, 50% and 97.5%, by using the 50th and 97.5th quantiles of the prevalence distributions in each pixel respectively.

### Validation Steps

We validated the implementation of the methodology by Touloupou et al [22]. by assessing the match between the geostatistical map and the combined weighted simulations from the transmission models. Formally, the performance of the method was assessed by comparing the observed and the estimated median of the baseline prevalence and population at each pixel, Supplementary Figures 8 and 9 in the Supplementary Material. To evaluate the projections from the models we compared the predictions for 2020 with the data reported in [23], Supplementary Figure 15.

## RESULTS

In this analysis, we combined a model-based geostatistical approach using historic data on prevalence of LF at the subnational level with the history of LF control programmes to fit 3 LF transmission models, which were then used for forward projections of LF incidence under different intervention scenarios.

**Figure 2.**
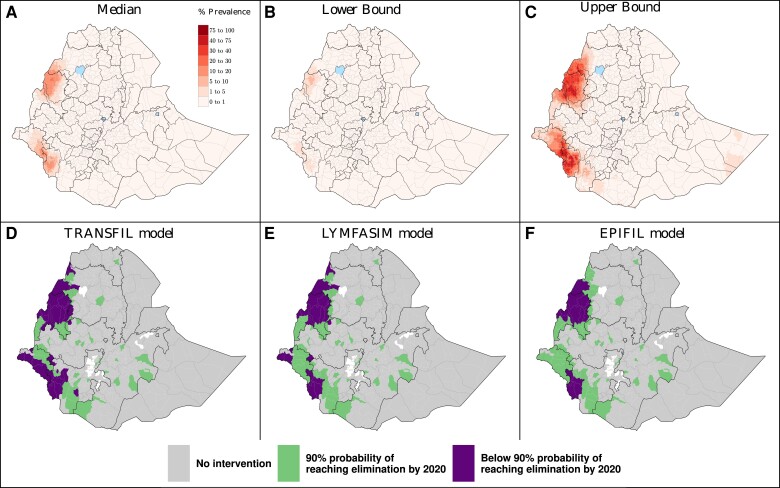
*A,* average (median) lymphatic filariasis mf prevalence prior to the start of MDA in Ethiopia (either 2008 or 2013) at pixel level (5 × 5 km square). *B, C*, Lower and upper bound mf prevalence representing the 2.5% and 97.5% quantiles, respectively. *D*, *E*, *F*, Predictions for reaching 1% mf prevalence by 2020 with 90% probability, under the “current intervention” strategy with TRANSFIL (*D*), LYMFASIM (*E*), and EPIFIL (*F*). Gray areas are considered non-endemic. Woredas in green have a probability above 90% of reaching EPHP (1% mf prevalence), whereas woredas in purple have a probability below 90% of reaching it. Abbreviations: EPHP, elimination as a public health problem; MDA, mass drug administration; mf, microfilaria.

Using this approach, we first predicted (median) mf prevalence across Ethiopia before intervention, together with estimates of lower (2.5%) and upper (97.5%) quantiles (Figure 2*A–C* ). Most of Ethiopia had a median mf prevalence below the 1% threshold for EPHP (93% of the pixels). By contrast, 90 woredas (using the 2013 woreda were estimated as endemic for LF. Of these, the highest average mf prevalence per pixel values were observed in the Southwest and Northwest regions. In particular, the pixel with the highest median mf value, located in the Northwest region, had an estimated prevalence of 30%.

Based on these pre-intervention predictions, we then incorporated individual histories of LF control for each woreda (each given a specific ITN coverage and MDA history) and projected the probability of each area achieving EPHP by 2020 while using a standard intervention (ie, finish five rounds of annual MDA with 65% coverage, if already started). Our results across the 3 transmission models suggested that EPHP (ie, reaching 1% mf prevalence) was likely to occur in most of Ethiopia by 2020 with the strategy being implemented, Figure 2*D*–*F* ; see Supplementary Figure 14 in the Supplementary Material for pixel level results. All 3 models suggested that 55–73 out of the 90 woredas estimated as endemic would have achieved elimination with a probability above 90% by 2020. Outcomes from the 3 models generally agree, with 17 woredas in the Northwest and Southwest regions estimated to have a probability below 90% of reaching elimination by 2020 across the 3 models.

Although our initial projections were made before 2020, the long timeframe of this project provided us with the opportunity to compare these models with 2020 data. Notably, the models were moderately good at predicting endemic woredas in 2020. For example, the 17 woredas in the Northwest and Southwest regions estimated to have a probability below 90% of reaching elimination by 2020 across the 3 models correlate well, although they do not completely overlap, with those requiring MDA in the 2021–2025 NTD Program from Ethiopia [23], Supplementary Figure 15 in Supplementary Materials. The main disagreement relates to those endemic woredas outside the Northwest and Southwest part of the country, which all 3 models consider would have reached elimination by 2020, but as reported in [23], they still require MDA.

**Figure 3.**
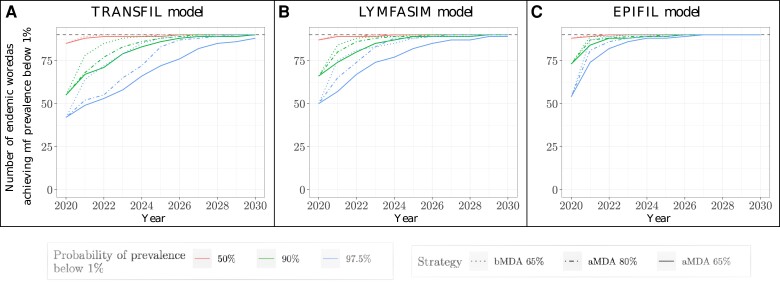
Number, out of the originally 90 endemic woredas, reaching the EPHP target (1% mf prevalence) over time, under different interventions starting in 2020: annual MDA rounds with 65% coverage (solid line), annual MDA with 80% achieved coverage (dot-dashed line), and biannual MDA with a 65% coverage achieved (dotted line). We report 3 certainty levels (ie, probabilities that a prevalence below 1% has been reached): 50% − red, 90% − green, and 97.5% − blue, across the three models *A*, TRANSFIL; *B*, LYMFASIM; and *C*, EPIFIL. Abbreviations: aMDA, annual mass drug administration; bMDA, biannual mass drug administration; EPHP, elimination as a public health problem; MDA, mass drug administration; mf, microfilaria.

Finally, we explored our models to extend projections beyond 2020. Given the original 2020 goals, a decision could have been made in 2020 to continue the current intervention strategy or expand the programme considering the 2030 roadmap, for example, by increasing the coverage achieved from 65% to 80%, or increasing MDA frequency from annual (aMDA) to biannual (bMDA). In order to consider how much the enhanced interventions would accelerate the achievement of the 2030 goals, we estimated the year that mf prevalence falls below the 1% pre-TAS threshold across the 90 woredas originally estimated as endemic under the three expanded programmes: 10 rounds of annual MDA 65% (aMDA 65%), 10 rounds of annual MDA 80% (aMDA 80%), or 10 years of biannual MDA 65% (bMDA 65%). We explored 3 values of certainty: 50%, 90%, and 97.5% probability of achieving elimination. The results are aggregated for each woreda and shown for each of the three models, Figure 3. Due to the uncertainty in the baseline prevalence, propagated through the simulations, the level of certainty (ie, confidence in the projections) will affect the number of woredas that are estimated to have achieved EPHP post-2020. Importantly, across all three models, all woredas were projected to reach EPHP by 2030, with a 90% probability, independently of the expanded programme adopted. In fact, most would be expected to reach it by 2025, the target year to reach EPHP set in the Ministry of Health strategic plan [23]. However, although deploying additional rounds of MDA with a 65% coverage (aMDA 65%) was likely sufficient to lead to elimination by 2030, the time to reach the goal can be accelerated by expanding the MDA programme to an increase in coverage (aMDA 80%) or an increase in the number of rounds (from annual to biannual, bMDA 65%). In our simulations, changing to biannual MDA with 65% coverage was projected to lead to a faster elimination timeline across endemic woredas than increasing annual MDA coverage to 80%.

## DISCUSSION

Our analyses show how integrating geostatistical mapping with transmission models and intervention histories can be used to project trends in LF prevalence at the subnational level in Ethiopia, over different time horizons and under a range of different control scenarios. Importantly, the models were moderately good at predicting 2020 prevalence, including by identifying woredas with a lower estimated probability of reaching elimination by 2020 that partially match those that were still endemic in that year, which therefore may require additional attention and interventions, Figure 2*D–F* , Supplementary Figure 15. Importantly, our approach also enabled us to explore how different enhanced interventions influence the achievement of the 2030 goals, Figure 3. Of these, although the standard strategy of annual MDA with 65% coverage was likely sufficient to achieve EPHP by 2030, alternative regimes such as biannual MDA or annual MDA with increased (80%) coverage both accelerated progress, leading to most woredas achieving EPHP with at least a 90% probability by 2024, Figure 3. These results reinforce insights that previous modelling studies have provided on the potential impact of increasing MDA coverage and/or frequency [6, 32], while tailoring them to the Ethiopian context.

Another important finding of our study is that while incorporating geostatistical prevalence data into our models enables sub-national projections, local context remains very important. In our models, coverage is a critical determinant for the success of interventions, but understanding true MDA coverage levels across different areas remains a key challenge. Furthermore, choosing the best intervention will depend on local operational challenges and on the costs of implementation, which are not considered here.

Another strength of our approach is that our simulations capture many sources of uncertainty, such as uncertainty in the prevalence and population size in each pixel, as well as variability in other model inputs and stochasticity in the transmission model. All 3 transmission models qualitatively agree, with EPIFIL being slightly more optimistic with respect to elimination by 2020 and the 2030 goals, while LYMFASIM and TRANSFIL have slightly more variation in the estimated prevalence for each IU, and thus require more years of treatment for achieving elimination across Ethiopia with a high certainty.

Despite these strengths, there are several limitations that impact our analyses. One of the key limitations is that our projections are based on historical baseline mf prevalence prior to the start of MDA in Ethiopia, Figure 1*A* . Although we used conservative assumptions, uncertainty in the baseline geospatial map can be a challenge for accurate predictions, which needs to be considered when interpreting model projections, Figure 2*B* and 2*C* . To further strengthen future projections, it will be important to incorporate more recent data, as well as updated intervention histories, into these models.

The adopted geostatistical approach allows the estimation of mf prevalence based on the ICT prevalence data while taking into account the different intervention histories across the country. This modeling approach is an extension of the geostatistical model used by Moraga et al [9]. which models mf prevalence independently of ICT and hence can only be applied when mf data have been collected. By incorporating the historical coverage of the different campaigns (MDA and bednets), the number of alternative future scenarios increases rapidly. Thus a series of pragmatic simplifications and assumptions needed to be made, such as the one mentioned above limiting the MDA coverage to either 65% or nothing. Interestingly, for the purpose of achieving the LF elimination goals, the current estimated prevalence values and future rounds of treatment appear to be the main determinant of whether EPHP will be achieved, rather than past ones. This has been highlighted in the literature [6] and therefore indicates that there is generally sufficient time to achieve the 2030 elimination goals if the appropriate campaigns are implemented.

One challenge for interpretation of the projections is that the 65% and 80% MDA coverages in the model are assumed to be achieved, which can be challenging in many settings (particularly reaching 80% coverage). Moreover, we assume a realistic value of compliance between rounds, which determines who gets treated and who is missed each round, based on previous work and standardized for the 2 stochastic models [6]. There is also a proportion of the population that is always missed (ie, never treated). However, there are generally limited data available on achieved coverage and quality of coverage (and the extent of compliance between rounds and proportion of individuals never treated), which will also have an effect on the assumptions regarding the histories of intervention. As our results are mainly driven by the coverage of future MDA strategies, post-campaign evaluations are recommended as a tool to verify that the target coverage is being achieved and that there are not large proportions of the population systematically being missed. Similarly, recent work highlights the importance of the elimination and post-elimination evaluation timelines for monitoring possible resurgence [11]. This may be further impacted by the effectiveness of bednets, which we considered constant throughout, but may be hampered by insecticide resistance, which has been reported in the region [33].

In our simulations, biannual MDA is slightly superior to annual MDA with 80% coverage in accelerating LF elimination across woredas. However, this advantage of biannual MDA over increased MDA coverage is dependent on model parameters, such as individuals reached each round, which as highlighted earlier are hard to inform; thus our result do not necessarily reflect whether an intervention is truly superior to the other one. The impact of biannual rounds of treatment could also potentially be overestimated, because our models do not account for logistical constraints in the implementation of the MDA campaigns or for changes in patterns of who is and who is not reached. This will be important as drug distribution requires time, and so does the drug itself to act [34]. For yearly rounds of treatment, these effects can be assumed to be fairly minor, however with biannual rounds these might become more important, and further work is needed to understand how they may influence disease transmission. Achieving this coverage in practice, whether 65% biannual or 80% annually, is very challenging in practice and will require “buy-in” from a range of stakeholders, from implementing partners, to local community champions and national and subnational authorities.

There are some additional limitations from our statistical approach, particularly in relation to capturing the spatial autocorrelation. In this framework, the results for each pixel are generated from an ensemble of all different model runs, but the pixels themselves are assessed independently. Spatial autocorrelation is imposed at the woreda level by assuming 100% rank correlation, that is, that all pixels within a woreda will be in the same percentile of their distribution.

Mapping has been a key strength for progress toward EPHP in Ethiopia [35, 36], and despite the limitations in our model, we show here how integrating geostatistical mapping with transmission models and program histories can help provide more usable predictions for policy makers at the appropriate spatial scale, which could help national level policymakers focus on areas that might need alternative approaches. Our framework is very flexible and allows us to make repeated use of the same simulations across similar locations to appropriately inform future interventions at a fine scale. The models characterize a large amount of uncertainty, and therefore it can be challenging to convey the most critical information in a simple manner, particularly to policy makers. Nonetheless, the analysis presented here is a proof-of-concept based on the successful programme in Ethiopia and could be implemented in other countries earlier in their LF programme life cycle, including to help identify areas where elimination is expected to be harder to achieve. Similarly, the approach may be expanded to other diseases, as more information becomes available on the spatial distribution of disease prevalence and appropriate transmission models are generated.

## Supplementary Data

Supplementary materials are available at *Clinical Infectious Diseases* online. Consisting of data provided by the authors to benefit the reader, the posted materials are not copyedited and are the sole responsibility of the authors, so questions or comments should be addressed to the corresponding author.

## Supplementary Material

ciae072_Supplementary_Data

## Notes

***Author contributions.*** Conceptualization: J. M. P., P. T., B. K., R. P., S. E. F. S., T. D. H., F, S. Data creation: J. M.P., P. T., B. K., E. G., H. S., J. C. Formal analysis: J. M. P., P. T., S. E. F. S., T. D. H. Funding acquisition: T. D. H. Investigation, methodology, and software: J. M. P., P. T., P. B., M. I., S. E. F. S., T. D. H. (TRANSFIL); E. G., R. P. (Geospatial model); M. S., R. C. R., E. M. (EPIFIL); P. K., S. J. d. V., W. A. S. (LYMFASIM). Project administration: J. M. P., P. T., H. F., T. D. H. Supervision: L. R., S. J. d. V., E. M., R. P., S. E. F. S., T. D. H., F. S. Validation: J. M. P., P. T. Visualization: J. M. P., P. T., T. D. H. Writing original draft: J. M. P., P. T. Writing, review and editing: all authors.

***Acknowledgments.*** The authors gratefully acknowledge the contributions of Claudio Nunes Alves in reviewing this article.

***Financial support.*** This work was supported by the Bill and Melinda Gates Foundation through the NTD Modelling Consortium (grant numbers OPP1186851, OPP1156227). This supplement is sponsored by funding of Professor T. Déirdre Hollingsworth's research by Li Ka Shing Foundation at the Big Data Institute, Li Ka Shing Centre for Health Information and Discovery, University of Oxford, and funding of the NTD Modelling Consortium by the Bill and Melinda Gates Foundation (grant number INV-030046).

***Supplement sponsorship.*** This article appears as part of the supplement “New Tools and Nuanced Interventions to Accelerate Achievement of 2030 Roadmap for Neglected Tropical Diseases,” sponsored by funding of Professor T. Déirdre Hollingsworth's research by the Li Ka Shing Foundation at the Big Data Institute, Li Ka Shing Centre for Health Information and Discovery, University of Oxford; and funding of the NTD Modelling Consortium by the Bill & Melinda Gates Foundation (INV-030046).
