# Supplementary material for: Subnational Projections of Lymphatic Filariasis Elimination Targets in Ethiopia to Support National Level Policy
Source: Clin Infect Dis. 2024 Apr 25;78(Suppl 2):S117–25. doi: 10.1093/cid/ciae072 (PMC11045027; doi:10.1093/cid/ciae072)
Supplement: ciae072_Supplementary_Data [file ciae072_supplementary_data.zip › Supplementary Geostatistical model.pdf]

Supplementary Material: Geostatistical model

J. M. Prada, P. Touloupou, et al.

September 29, 2023

Let Y_i denote the random variable associated with the number of LF cases out of n_i, that are detected using the immuno-chromatographic test (ICT) at geographical location x_i, for $i = 1, \ldots, n$.

We then assume that, conditionally on a Gaussian process $S(x_i)$ and Gaussian noise Z_i, the Y_i are mutually independent Binomial variables with probability of a positive ICT test given by (Irvine et al., 2016)

$$p(x_i) = p_0(1 - \exp\{-\lambda(x_i)\}) \tag{1}$$

where p_0 is the ICT sensitivity and $\lambda(x_i)$ is the mean number of worms which we model as a log-linear regression, i.e.

$$\log\{\lambda(x_i)\} = \beta + S(x_i) + Z_i. \tag{2}$$

The random effects $S(x_i)$ and Z_i account for the large- and small-scale spatial variation in the mean number of worms, respectively. We model $S(x)$ as an isotropic and stationary Gaussian process with mean 0 and covariance function

$$\text{cov}\{S(x_i), S(x_j)\} = \sigma^2 \exp\{-\|x_i - x_j\|/\phi\}$$

where σ^2 is the variance of $S(x)$, $\| \cdot \|$ is the Euclidean distance and ϕ is a scale parameter which regulates how fast the spatial correlation decays to 0 for increasing distance between locations. Finally, we assume that the Z_i are independent and identically distributed Gaussian variables with mean 0 and variance τ^2.

In modelling ICT prevalence, we assume a specificity of 100% and a sensitivity $p_0 = 97\%$ (Chandrasena et al., 2002; Weil & Ramzy, 2007).

We carry out estimation of the model parameters using the Monte Carlo maximum likelihood (Christensen, 2004).

Spatial prediction of micro-filariae prevalence

To predict micro-filariae (MF) prevalence at a given location x, we proceed as follows.

1. Obtain predictive samples of the mean number of worms, given by (2), by simulation from the distribution of $\lambda(x)$ given the data $Y_i = y_i$.

2. Let $\lambda_{(j)}(x)$ denote the j-th Monte Carlo sample, for $j = 1, \ldots, B = 10000$ for the mean number of worms at x obtained in the previous step. For each sample we then estimate MF prevalence using the following expression (Irvine et al., 2016)

$$q_{(j)}(x) = 1 - \exp\{-\alpha\{1 - \exp(-\lambda_{(j)}(x)\}\}, \tag{3}$$

   where α is the detection rate which we set to 0.2 (Hairston & de Meillon, 1968).

3. Use the Monte Carlo samples $q_{(j)}(x)$ to estimate MF prevalence using the predictive mean

$$\hat{q}(x) = \frac{1}{B} \sum_{j=1}^{B} q_{(j)}(x)$$

   and to compute relevant summaries of uncertainty, e.g. standard errors and confidence intervals.

To simulate predictive samples in first step of the above algorithm, we use a Laplace sampling technique described in detail in Section 2.1 of Giorgi & Diggle (2017).

References

Chandrasena, N., Premaratna, R., Abeyewickrema, W. & Silva, N. (2002). Evaluation of the ict whole-blood antigen card test to detect infection due to wuchereria bancrofti in sri lanka. *Transactions of the Royal Society of Tropical Medicine and Hygiene* **96**, 60–3.

Christensen, O. F. (2004). Monte Carlo maximum likelihood in model-based geostatistics. *Journal of Computational and Graphical Statistics* **3**, 702–718.

Giorgi, E. & Diggle, P. J. (2017). PrevMap: An R package for prevalence mapping. *Journal of Statistical Software* **78**, 1–29.

Hairston, N. G. & de Meillon, B. (1968). On the inefficiency of transmission of wuchereria bancrofti from mosquito to human host. *Bulletin of the World Health Organization* **38**, 935–941.

Irvine, M. A., Njenga, S. M., Gunawardena, S., Njeri Wamae, C., Cano, J., Brooker, S. J. & Deirdre Hollingsworth, T. (2016). Understanding the relationship between prevalence of microfilariae and antigenaemia using a model of lymphatic filariasis infection. *Transactions of The Royal Society of Tropical Medicine and Hygiene* **110**, 118–124.

Weil, G. J. & Ramzy, R. M. R. (2007). Diagnostic tools for filariasis elimination programs. *Trends in parasitology* **23 2**, 78–82.
