# Supplementary material for: Subnational Projections of Lymphatic Filariasis Elimination Targets in Ethiopia to Support National Level Policy
Source: Clin Infect Dis. 2024 Apr 25;78(Suppl 2):S117–25. doi: 10.1093/cid/ciae072 (PMC11045027; doi:10.1093/cid/ciae072)
Supplement: ciae072_Supplementary_Data [file ciae072_supplementary_data.zip › Supplementary Transmission Models_updated.docx]

# Supplementary Material: Transmission Models

Prada, Touloupou *et al.* 2023

Index

EPIFIL model description and methods 2

LYMFASIM model description and methods 10

TRANSFIL model description and methods 21

Validation of model integration 29

Historical intervention scenarios 31

Pixel-level 2020 projections 34

Lymphatic filariasis elimination status in 2020 35

EPIFIL model description and methods

## The mathematical model of LF transmission dynamics

We employed a genus specific mosquito-vectored transmission model of LF to carry out the modelling work in this study 1-7. Briefly, the state variables of this hybrid coupled partial differential and differential equation model vary over age (*a*) and/or time (*t*), representing changes in the pre-patent worm burden per human host adult worm burden per human hostthe microfilariae (mf) level in the human host modified to reflect infection detection in a 1 mL blood sample the average number of infective L3 larval stages per mosquito (*L*), and a measure of immunity developed by human hosts against L3 larvae. The state equations comprising this model are:

The above equations involve partial derivatives of four state variables (*P* - pre-patent worm load; *W* - adult worm load; *M* - microfilaria intensity; *I* - immunity to acquiring new infection due to the pre-existing total worm load where *WT = W(a,t) + P(a,t)*). Given the faster time scale of infection dynamics in the vector compared to the human host, the infective L3-stage larval density in mosquito population is modelled by an ordinary differential equation essentially reflecting the significantly faster time-scale of the infection dynamics in the vector hosts. This allows us to make the simplifying assumption that the density of infective stage larvae in the vector population reaches a dynamic equilibrium (denoted by *L**) rapidly1, 2, 5, 8, 9. This basic coupled immigration-death structure of the model as well as its recent extensions has been extensively discussed previously1-3, 5, 8, 9. The effects of worm patency are captured by considering that at any time *t*, human individuals of age less than or equal to the pre-patency period, *τ*, will have no adult worms or mf, and the rate at which pre-patent worms survive to become adult worms in these individuals at *a > τ* is given by . The term enables us to account for the different establishment and development rates of the incoming L3-stage larvae as adult worms depending on the genus of mosquito vectors as expressed below:

for mosquitoes of *Anopheline* genus,

for mosquitoes of the Culicine genus.

In the above, is the shape parameter of the negative binomial distribution on the mf uptake whereas r and are respectively the rate of initial increase and the maximum level of L3 larvae. See Table 1 for the description of all the model parameters and functions.

**Table S1** – Description of EPIFIL model parameters and functions.

| **Parameter** | **Definition (*units*)** | **Range** | **Refs** |
| --- | --- | --- | --- |
| ***λ*** | Number of bites per mosquito (*per month*) | [5, 15] | 1, 2, 5, 10, 11 |
| ***τ*** | Pre-patency period | [1, 9] | 12 |
| ***s*** | Proportion of female worms | 0.5 | - |
| ***μ*** | The worm mortality rate (*per month*) | [0.008, 0.018] | 1, 2, 5, 13-16 |
| ***α*** | Production rate of microfilariae per worm (*per month*) | [0.25, 1.5] | 1, 2, 5, 17 |
| ***γ*** | The death rate of the microfilariae (*per month*) | [0.08, 0.12] | 1, 5, 15, 17 |
| ***g*** | Proportion of mosquitoes which pick up infection when biting an infected host | [0.251, 0.485] | 1, 5, 18 |
| ***κ*** | Maximum level of L3 given mf density | [3, 5] | 1, 5 |
| ***k0*** | The basic location parameter of negative binomial distribution used in aggregation parameter  () | [0.000036, 0.000775] | 1, 5, 19, 20 |
| ***δ*** | Immunity waning rate (*per month*) | [0.001, 0.01] | 1, 5 |
| ***V*** | Vector population size | [25000, 100000] | data |
| ***H*** | Human population size | data | data |
| ***kLin*** | The linear rate of increase in the aggregation parameter defined above | [0.00000024, 0.282] | 1, 5, 19, 20 |
| ***σ*** | Death rate of mosquitoes (*per month*) | [1.5, 8.5] | 1, 5, 20 |
| ***ψ1*** | Proportion of L3 leaving mosquito per bite | [0.1, 0.8] | 17 |
| ***ψ2*** | The establishment rate1 | [0.00003, 0.00364] | 1, 2, 5, 21 |
| ***HLin*** | A threshold value used in *h(a)* to adjust the rate at which individuals of age *a* are bitten: linear rise from 0 at age zero to 1 at age *HLin* in years. | [240, 360] months | 1, 5, 9 |
| ***r*** | Gradient of mf uptake2 | [0.04, 0.25] | 1, 5 |
| ***c*** | Strength of acquired immunity | [0.015, 0.025] | 1, 5 |
| ***IC*** | Strength of immunosuppression3 | [0.5, 5.5] | 1, 5 |
| ***SC*** | Slope of immunosuppression function4  (*per worm/month*) | [0.01, 0.20] | 1, 5 |
| ***Intervention-related parameters*** | | | |
| ***ω*** | Worm killing efficacy of drug (instantaneous) | dependent on drug regimen | 3 |
| ***ε*** | Microfilariae killing efficacy of drug (instantaneous) | dependent on drug regimen | 3 |
| ***δreduc*** | Reduction in the worm’s fecundity over a period of time *p* due to drug | dependent on drug regimen | 3 |
| ***p*** | A time period during which the drug remains efficacious in reducing the fecundity of the surviving adult worms | dependent on drug regimen | 3 |
| ***C*** | Percentage of the population administered the drug | data | data |
| ***MBRVC*** | Vector control (VC) modifies ()where, withfor when VC is implemented, otherwise. | data and estimates | 19, 20 |
| **Description** | **Mathematical expressions of the functions** | **Parameters** |  |
| Probability that an individual is of age *a* ***π(a)*** |  | Human age *a* in month, *A0* and *B0* estimated from country demographic data | 1, 5, 9 |
| Larvae establishment rate (modified by acquired immunity) ***Ω(a,t)*** |  | 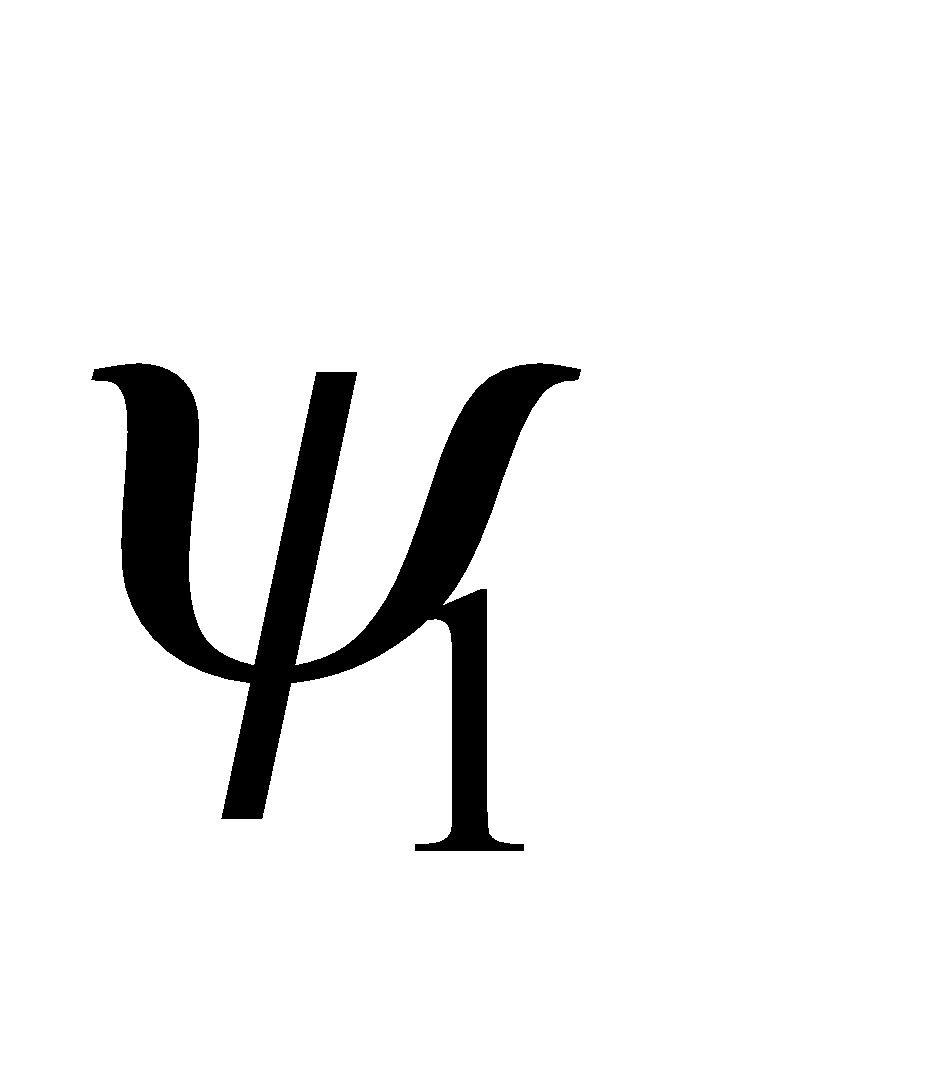- proportion of L3 leaving mosquito per bite; 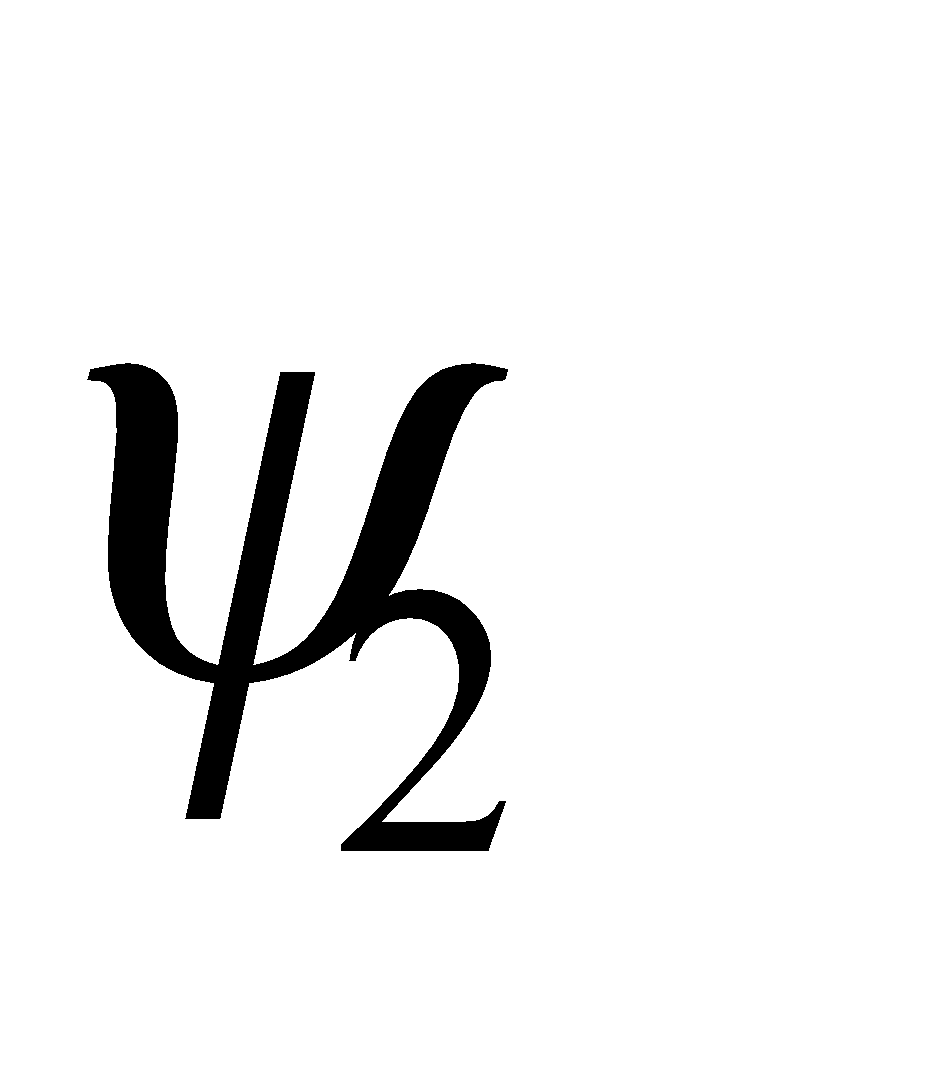- the establishment rate1 | - |
| Adult worm mating probability ***ϕ(W,k)*** |  | *k* – negative binomial aggregation parameter | 2, 5, 22 |
| Immunity to larval establishment ***g1(I)*** |  | *c* – strength of immunity to larval establishment | 1, 5 |
| Host immunosuppression  ***g2(WT)*** |  | *IC* – strength of immunosuppression;  *SC* – slope of immunosuppression | 1, 5 |

1The proportion of L3-stage larvae infecting human hosts that survive to develop into adult worms2.

2The gradient of mf uptake *r* is a measure of the initial increase in the infective L3 larvae uptake by vector as *M* increases from 02, 9.

3 The facilitated establishment rate of adult worms due to parasite-induced immunosuppression in a heavily infected human host

4 The initial rate of increase by which the strength of immunosuppression is achieved as *W* increases from 023.

# Note MBR (monthly biting rate) serves as an input to initialize the model, measured as mosquito bites per person per month, the value of which may be obtained from entomological surveys conducted in study sites. In the absence of the observed MBR value, the model has been adapted to estimate it from the community-level mf prevalence data.

**Model implementation**

*Parameter selection and simulation procedure*

We employed the Bayesian Melding (BM) procedure to calibrate and estimate the LF models from field data, as outlined in detail in our previous work 2, 5-7. Typically, we begin the procedure by first using the known or assignment of a uniform range for parameter values to generate distributions of parameter priors. We then randomly sample with replacement from these prior distributions to generate 200,000 parameter vectors, which are run using the annual biting rate (ABR) values, if given, for a site to generate model outputs. The model outputs are then melded with age-stratified mf prevalence data by calculating binomial log-likelihoods for each parameter vector. In the resampling step of the BM method, a Sampling-Importance-Resampling (SIR) algorithm is used to perform 500 draws with replacement from the pool of parameter vectors generated as above, with probabilities proportional to their relative log likelihood values. This step selects the parameter vectors which best describe the given mf age-prevalence data. These resampled parameter vectors are then used to generate distributions of variables of interest from the fitted model (eg. age-prevalence curves, worm breakpoints, and infection trajectories following treatments).

Here, we modified our standard Monte-Carlo BM framework for model discovery 2, 5-7, 24 to provide simulations for the chosen scenarios in Ethiopia. The explicit aim was to generate at least N = 100,000 parameter vectors which resulted in a uniform distribution of overall mf prevalence values 0-60% at the time of simulation weighting (2008 or 2013 for a given scenario). For all simulations, the Anopheline-transmitted LF model was used. For each of the four regions considered, we first randomly sampled n = 100 000 parameter vectors from the assigned uniform parameter priors. We then simulated the described bednet scenarios from 2005 to the time of weighting and calculated the predicted mf prevalence at the time of weighting. Those parameter vectors whose outputs produced mf prevalence values between 0-60% were accepted while all others are rejected. This sampling and acceptance/rejection procedure was repeated until the total number of accepted parameter vectors (N) was greater than or equal to 100,000. To ensure that a uniform posterior distribution of prevalences was generated by this approach, model selection was done such that an equal number of models were selected from 5% prevalence bins (ex. 0-5%, 5-10%, etc). To avoid accepting extremely small prevalences as non-zero, we set a prevalence value of 1 x 10-6 % as our lower bound. The N posterior parameter vectors were then used to simulate the impacts of MDA and vector control interventions.

## Modeling intervention by mass drug administration

Intervention by mass drug administration was modeled based on the assumptions that anti-filarial treatment with a combination drug regimen acts by killing certain fractions of the populations of adult worms and microfilariae instantly after the drug administration25. These effects are incorporated into the basic model by calculating the population sizes of worms and microfilariae as follows:

where *dt* is a short time period since the *i*th MDA was administered. During this short time interval, a given proportion of adult worms and microfilariae are instantly removed. The parameters *ω* and *ε* are drug killing efficacy rates for the two life stages of the parasite while the parameter *C* represents the MDA coverage. Apart from instantaneous killing of microfilariae, the drug continues to kill the newly reproduced mf by any surviving adult worms at a rate *δreduc* for a period of time, *p*. We model this effect as follows:

We simulated LF intervention by running the model with fixed values of *ω*, *ε*, *δreduc*, and *p* for MDA coverage levels given by the scenarios. The first MDA round was implemented in the model by affecting the population sizes of worms and microfilariae from the baseline estimates, and then the intervention is simulated forward in time for a number of years, with subsequent MDA rounds implemented annually or biannually.

## Modeling intervention by Vector Control

In addition to MDA, we also modeled the added effect of long lasting insecticidal nets (LLINs) as described previously 6. The impact of LLINs with three main actions against mosquito biting was modelled: 1) deterrence from entering the home (efficacy *η1*), 2) inhibition of their ability to feed on humans (efficacy *η2*), and 3) killing them (efficacy *η3*) 26,27.To capture these effects, which decay over time as the larvicide efficacy declines exponentially at rate *Λ*, we adjust the term *V/H* to be appropriately modified according to the population coverage for LLINs (*CLLIN*)*:*

**References**

1. Gambhir, M. & Michael, E. Complex ecological dynamics and eradicability of the vector borne macroparasitic disease, lymphatic filariasis. *PLoS One* **3**, e2874 (2008).

2. Gambhir, M. *et al*. Geographic and ecologic heterogeneity in elimination thresholds for the major vector-borne helminthic disease, lymphatic filariasis. *BMC biology* **8**, 1 (2010).

3. Michael, E. *et al*. Mathematical modelling and the control of lymphatic filariasis. *The Lancet infectious diseases* **4**, 223-234 (2004).

4. Michael, E., Malecela-Lazaro, M. N., Kabali, C., Snow, L. C. & Kazura, J. W. Mathematical models and lymphatic filariasis control: endpoints and optimal interventions. *Trends Parasitol.* **22**, 226-233 (2006).

5. Singh BK and Bockarie MJ and Gambhir M and Siba PM and Tisch DJ and Kazura J and others. in *Sequential Modelling of the Effects of Mass Drug Treatments on Anopheline-Mediated Lymphatic Filariasis Infection in Papua New Guinea* (PLoS One, 2013).

6. Singh, B. K. & Michael, E. Bayesian calibration of simulation models for supporting management of the elimination of the macroparasitic disease, Lymphatic Filariasis. *Parasit vectors* **8**, 1-26 (2015).

7. Michael, E. & Singh, B. K. Heterogeneous dynamics, robustness/fragility trade-offs, and the eradication of the macroparasitic disease, lymphatic filariasis. *BMC medicine* **14**, 1 (2016).

8. Chan, M. S. *et al*. Epifil: a dynamic model of infection and disease in lymphatic filariasis. *Am. J. Trop. Med. Hyg.* **59**, 606-614 (1998).

9. Norman, R. *et al*. EPIFIL: the development of an age-structured model for describing the transmission dynamics and control of lymphatic filariasis. *Epidemiol. Infect.* **124**, 529-541 (2000).

10. Rajagopalan, P. Population dynamics of culex pipiens fatigans, the filariasis vector, in pondicherry: influence of climate and environment. *Proc Ind Nat Science Acad B* **46**, 745-752 (1980).

11. Subramanian, S., Manoharan, A., Ramaiah, K. D. & Das, P. K. Rates of acquisition and loss of Wuchereria bancrofti infection in Culex quinquefasciatus. *Am. J. Trop. Med. Hyg.* **51**, 244-249 (1994).

12. Scott, A. L. & Nutman, T. Lymphatic-dwelling filariae. *Lymphatic filariasis.*, 5-39 (2000).

13. Vanamail, P., Subramanian, S., Das, P. K., Pani, S. P. & Rajagopalan, P. K. Estimation of fecundic life span of Wuchereria bancrofti from longitudinal study of human infection in an endemic area of Pondicherry (south India). *Indian J. Med. Res.* **91**, 293-297 (1990).

14. Evans, D. B., Gelband, H. & Vlassoff, C. Social and economic factors and the control of lymphatic filariasis: a review. *Acta Trop.* **53**, 1-26 (1993).

15. Ottesen, E. & Ramachandran, C. Lymphatic filariasis infection and disease: control strategies. *Parasitology Today* **11**, 129-130 (1995).

16. Vanamail, P. *et al*. Estimation of the fecund life span of Wuchereria bancrofti in an endemic area. *Trans. R. Soc. Trop. Med. Hyg.* **90**, 119-121 (1996).

17. Hairston, N. G. & de Meillon, B. On the inefficiency of transmission of Wuchereria bancrofti from mosquito to human host. *Bull. World Health Organ.* **38**, 935-941 (1968).

18. Subramanian, S. *et al*. The relationship between microfilarial load in the human host and uptake and development of Wuchereria bancrofti microfilariae by Culex quinquefasciatus: a study under natural conditions. *Parasitology* **116**, 243-255 (1998).

19. Subramanian, S., Pani, S., Das, P. & Rajagopalan, P. Bancroftian filariasis in Pondicherry, south India: 2. Epidemiological evaluation of the effect of vector control. *Epidemiol. Infect.* **103**, 693-702 (1989).

20. Das, P. *et al*. Bancroftian filariasis in Pondicherry, south India–epidemiological impact of recovery of the vector population. *Epidemiol. Infect.* **108**, 483-493 (1992).

21. Ho, B. C. & Ewert, A. Experimental transmission of filarial larvae in relation to feeding behaviour of the mosquito vectors. *Trans. R. Soc. Trop. Med. Hyg.* **61**, 663-666 (1967).

22. May, R. M. Togetherness among schistosomes: its effects on the dynamics of the infection. *Math. Biosci.* **35**, 301-343 (1977).

23. Duerr, H., Dietz, K. & Eichner, M. Determinants of the eradicability of filarial infections: a conceptual approach. *Trends Parasitol.* **21**, 88-96 (2005).

24. Poole, D. & Raftery, A. E. Inference for deterministic simulation models: the Bayesian melding approach. *Journal of the American Statistical Association* **95**, 1244-1255 (2000).

25. Michael, E. et al., Quantifying the value of surveillance data for improving model predictions of lymphatic filariasis elimination. *Plos NTD, 12*(10), e0006674 (2018).

26. Griffin, J. T. *et al*. Reducing Plasmodium falciparum malaria transmission in Africa: a model-based evaluation of intervention strategies. *PLoS Med* **7**, e1000324 (2010).

27. Okumu, F. O. & Moore, S. J. Combining indoor residual spraying and insecticide-treated nets for malaria control in Africa: a review of possible outcomes and an outline of suggestions for the future. *Malaria journal* **10**, 1 (2011).

LYMFASIM model description and methods

## Description of the mathematical model

LYMFASIM1,2 is a stochastic individual-based model for lymphatic filariasis (LF). It is a specific model variant within WORMSIM, a generalized framework for modelling transmission and control of helminth infections in humans3,4. LYMFASIM simulates the life histories of individual people and individual worms in a community, and the effects of interventions (e.g. mass drug administration, integrated vector management, bednet use) on transmission and morbidity, while taking into account the human demography and the complexities of helminth transmission. The model has been described elsewhere and has been applied to support decision making on control and elimination of lymphatic filariasis in different settings1,2,5–13.

Mass drug administration (MDA) is simulated by specifying the exact timing of the treatment rounds (year, month), the efficacy of the applied treatment regimen, the achieved coverage level, and compliance patterns. LYMFASIM assumes that a fraction of people never participates in MDA (e.g. systematic refusal, related to chronic illness). In addition, LYMFASIM allows the relative compliance to vary between age and sex groups; this mechanism captures transient contra-indications for MDA (e.g. exclusion of young children and pregnant women) and other age- and sex-related behavioral factors driving participation in MDA. Lastly, each individual has a personal inclination to participate in MDA, which is considered as a lifelong property. A stochastic process eventually defines for each individual whether they are treated in a given round, depending on the calculated probability.

## Parameter quantification and simulation methods for this study

To simulate LF transmission by Anopheles in Ethiopia we used previously derived model parameterization for Africa8. Parameter values are listed in Table S2. Assumptions and parameters related to control strategies and treatment efficacy are listed in Table S3. The MDA timing and coverage simulated was 5 annual rounds set to 65% coverage. The monthly biting rate (mbr) and exposure heterogeneity parameter (k) were varied to generate simulations across a wide range of mf prevalences at baseline, measured in the total population (all ages). The density plots, Figures S2-S4, illustrate the parameter space areas from the Anopheles-simulations that were in the 0 to 60% mf prevalence range at baseline, 100 000 runs.


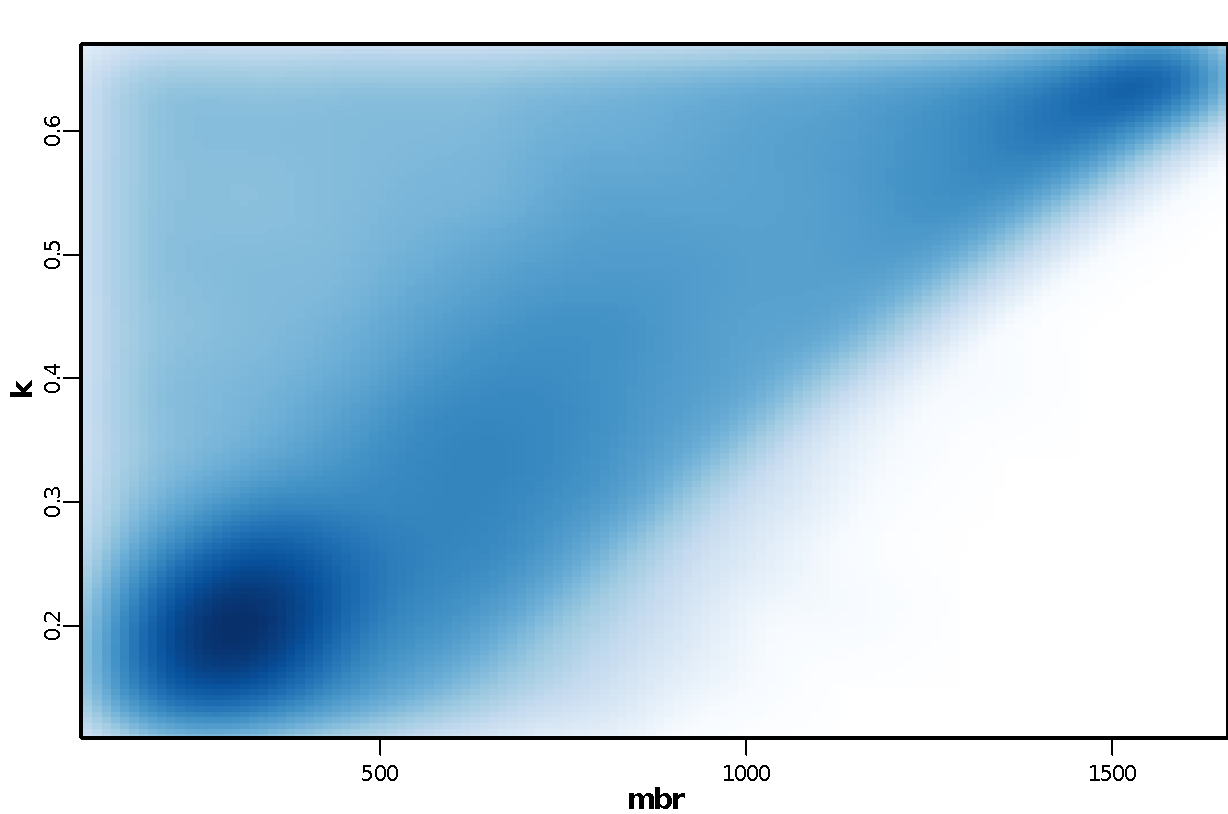


**Figure S1** – Monthly biting rate, mbr, against exposure heterogeneity parameter k, for the Anopheles simulations. The density plot illustrates the parameter space areas from the simulations that were in the 0 to 60% mf prevalence range at baseline. Dark blue areas denote more common parameter values.


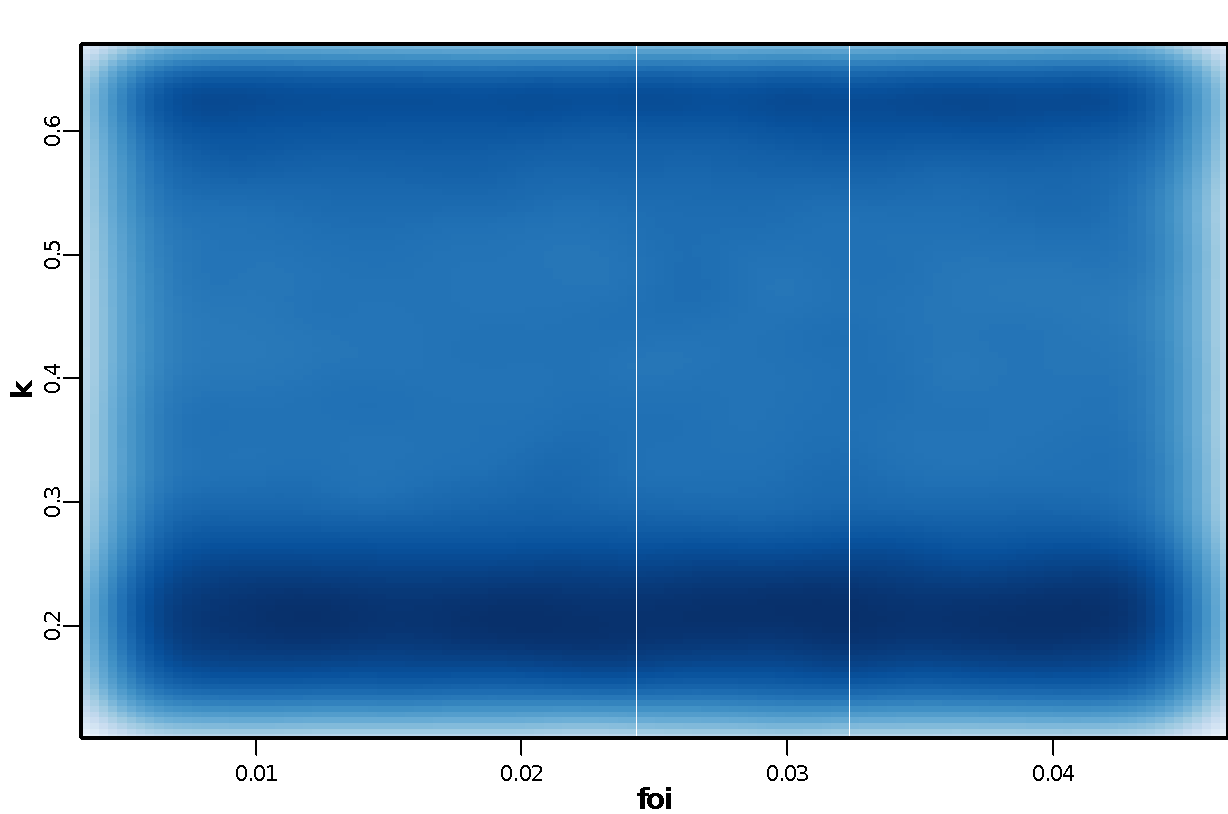


**Figure S2** – Force of infection, foi, against exposure heterogeneity parameter k, for the Anopheles simulations. As above, the density plot illustrates the parameter space areas from the simulations that were in the 0 to 60% mf prevalence range at baseline. Dark blue areas denote more common parameter values.


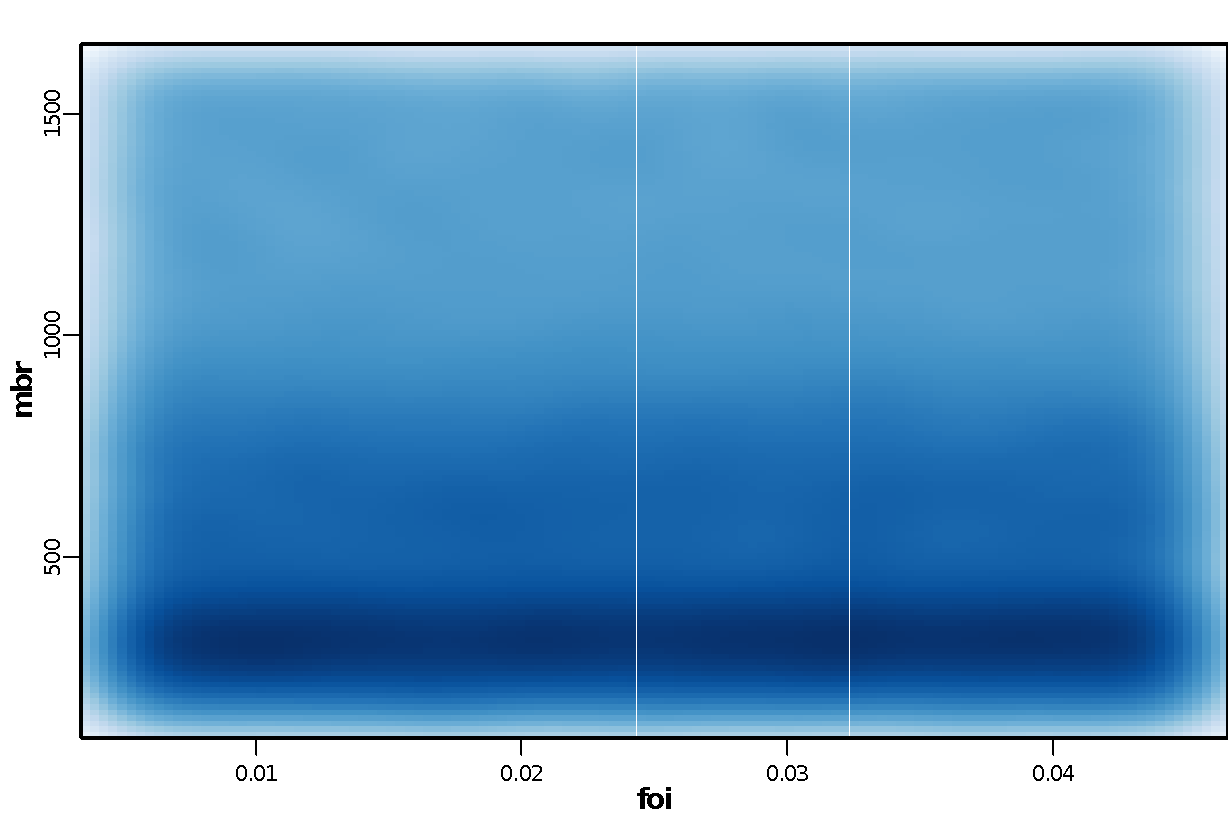


**Figure S3** – Force of infection, foi, against monthly biting rate, mbr, for the Anopheles simulations. As above, the density plot illustrates the parameter space areas from the simulations that were in the 0 to 60% mf prevalence range at baseline. Dark blue areas denote more common parameter values.

## Version and code availability

## For this paper, we used WORMSIM version 2.58Ap25. The code for this version of LYMFASIM used in this paper is available from [https://gitlab.com/erasmusmc-public-health/wormsim.previous.versions](https://eur02.safelinks.protection.outlook.com/?url=https%3A%2F%2Fgitlab.com%2Ferasmusmc-public-health%2Fwormsim.previous.versions&data=02%7C01%7Cj.prada@surrey.ac.uk%7C12ece7a7e8574e707e6f08d73825b19d%7C6b902693107440aa9e21d89446a2ebb5%7C0%7C0%7C637039606864181918&sdata=84hpL5TX%2BYizbL775n2bG9jihTBqT8t00jySoQT9mQg%3D&reserved=0).

**Table S2** – LYMFASIM input: probability distributions, functions and parameter values for simulating transmission of bancroftian filariasis by Anopheles mosquitoes in Africa

| **Parameter description** | **Anopheles model** | | **Source / remarks** |
| --- | --- | --- | --- |
| **Human demography** |  |  |  |
| Cumulative survival, by age | Age | Survival | Modified from ref8¸to match the population composition in Ethiopia; |
|  | 0 | 1 |
|  | 5 | 0.800 |
|  | 15 | 0.790 |
|  | 20 | 0.755 |
|  | 25 | 0.737 |
|  | 30 | 0.723 |
|  | 35 | 0.654 |
|  | 40 | 0.605 |
|  | 45 | 0.560 |
|  | 50 | 0.506 |
|  | 60 | 0.487 |
|  | 70 | 0.305 |
|  | 80 | 0.155 |
|  | 99 | 0.000 |
|  |  |  |  |
| Fertility rate per woman, by age | Age | Fertility rate | Fixed, as in 8; |
|  | 0 | 0 |
|  | 5 | 0 |
|  | 15 | 0 |
|  | 20 | 0.116 |
|  | 25 | 0.230 |
|  | 30 | 0.245 |  |
|  | 35 | 0.207 |  |
|  | 40 | 0.147 |  |
|  | 45 | 0.077 |  |
|  | 50 | 0.031 |  |
|  | 60 | 0 |  |
|  | 70 | 0 |  |
|  | 80 | 0 |  |
|  | 99 | 0 |  |
|  |  |  |  |
| Initial population | age | Male/females | N/A |
|  | 5 | 42/42 |  |
|  | 15 | 63/63 |  |
|  | 20 | 26/26 |  |
|  | 25 | 22/22 |  |
|  | 30 | 20/20 |  |
|  | 35 | 17/17 |  |
|  | 40 | 14/14 |  |
|  | 45 | 11/11 |  |
|  | 50 | 9/9 |  |
|  | 60 | 14/14 |  |
|  | 70 | 9/9 |  |
|  | 80 | 3/3 |  |
|  | 99 | 1/1 |  |
|  |  |  |  |
| Maximum population size | Varied according to population size distribution defined elsewhere in this manuscript | | Figure S1 |
| Proportion removed when maximum population size is reached | 5% |  | N/A |
|  |  |  |  |
| **Exposure** |  |  |  |
| External force-of-infection at start of burn-in period | 2 |  | N/A |
| Duration of external force-of-infection at start of burn-in period | 2 years |  | N/A |
| External force-of-infection (foi) during burn-in period | Sampled from a uniform distribution between 0.005 and 0.045; assumed to decline gradually after the introduction of mass treatment in the simulated population | | N/A |
| Average mosquito biting rate for adult men (mbr), for a relative biting rate of 1 | Varied between runs, as specified in figure S2 | | N/A |
| Seasonal variation in biting rate | No | | N/A |
| Variation in exposure by age (no difference assumed between sexes) | 0 at birth, linearly increasing to reach 1 at the age of 20 and constant at 1 from this age onwards | | Slightly adjusted from 2 for Africa8; |
| Probability distribution describing variation in the individual exposure index, due to personal factors (fixed through life) given age and sex | Gamma distribution with mean 1.0 and shape (=rate) varied uniformly with the range [0.1-1] (see Figure S2) | | Gamma distribution is assumed as in8; shape/rate parameter varied |
|  |  |  |  |
| **Parasite dynamics within host** |  |  |  |
| Success ratio | 0.00088 |  | Previously estimated by fitting to data8,2 |
| Anti-L3 immunity | Not included in the model, by assuming that the strength and duration of the immunological memory are zero | | Assumed not to play a role8; |
| Anti-fecundity immunity: | Not included in the model, by assuming that the strength and duration of the immunological memory are zero | | Assumed8,2 |
| Average worm lifespan | 10 years on average; varied according to a Weibull distribution with shape 2 | | Previously estimated by fitting to data2 |
| Duration of immature stage of the parasite in human host | Constant, 8 months | | Fixed, based on 14 |
| No. of Mf produced per female parasite per month per 20 ml peripheral blood in the absence of immune reactions and in the presence of at least 1 male worm | 0.58 |  | Previously estimated by fitting to data2 |
| Monthly survival of the microfilariae, fraction | 0.9 |  | Fixed, based on15 |
| Association between worm age and mf production rate | mf production independent of worm age | | Assumed |
| Polygamy (all female worms produce mf in the presence of at least one male worm) | Yes (male potential 1000) | | Assumed |
| Mating cycle (number of months a female can produce mf with one insemination) | 1 | | Assumed |
|  |  | |  |
| **Uptake of infection by the vector** |  | |  |
| Functional relationship |  | | Fixed, based on 8,16 |
|  | a | 1.666 |  |
|  | b | 0.027 |  |
|  | c | 1.514 |  |
| Transmission probability (v), fraction of the L3 larvae, resulting from a single blood meal, that is released by a mosquito | 0.1 |  | Fixed, as in8 |
| **Other** |  |  |  |
| Start year of simulation period (burn-in period runs from this start-year to the moment of first intervention) | 1850 |  | N/A |
|  |  |  |  |
| **Surveillance** |  |  |  |
| Timing of surveys | Yearly, at the start of a calendaryear from 2005-2030 onwards (preceding mass treatment, when it occurs in the same year) | | N/A |
| Volume of blood examined for mf | 60 μL | | N/A |
| Variability in observed number of mf in one 20 μl blood smear | Negative binomial distribution with k=0.33 | | Previously estimated for 20 μL blood by fitting to data2 |
| Variation between worms in their contribution to measured mf count (dispersal factor) | Constant (no variation) | | Assumed |
|  |  |  |  |
| **Morbidity** |  |  |  |
| not applicable |  |  |  |
| no excess mortality due to disease |  |  |  |

**Table S3** – LYMFASIM assumptions related to interventions scenarios (MDA and bednet use is pixel specific and detailed below).

| **Parameter** | | **Values** | | **Source** |
| --- | --- | --- | --- | --- |
| **Mass treatment** | | | | |
| *Relative compliance by age and sex* | | | |  |
| age-group | | Males | Females | Based on unpublished OCP data |
| 0-4b | | 0[[1]](#footnote-1) | 0b |
| 5-9 | | 0.75 | 0.5 |
| 10-14 | | 0.8 | 0.7 |
| 15-19 | | 0.8 | 0.74 |
| 20-29 | | 0.7 | 0.65 |
| 30-49 | | 0.75 | 0.7 |
| 50+ | | 0.8 | 0.75 |
|  | | | | |
| **Drug treatment** | | | | |
| Fraction malabsorption (no effect) | | 0% | |  |
| Efficacy ivermectin + albendazole | | | |  |
|  | Proportion of adult worms killed per treatment, average | 35% | |  |
|  | Duration of temporary reduction in female reproductive capacity, average | 9 months[[2]](#footnote-2) | |  |
|  | Permanent reduction in female worm reproductive capacity, average | 0% | |  |
|  | Variability in effect of treatment on adult worms | Not applicable (assumed constant, 1) | |  |
|  | Fraction of mf surviving per treatment | 1% | |  |
| Efficacy diethylcarbamazine + albendazole | | | |  |
|  | Proportion of adult worms killed per treatment, average | 55% | |  |
|  | Duration of temporary reduction in female reproductive capacity, average | N/A | |  |
|  | Permanent reduction in female worm reproductive capacity, average | 0% | |  |
|  | Variability in effect of treatment on adult worms | Not applicable (assumed constant, 1) | |  |
|  | Fraction of mf surviving per treatment | 5% | |  |

**References**

1 Plaisier AP, Subramanian S, Das PK (1998). The LYMFASIM simulation program for modeling lymphatic filariasis and its control. Methods Inf Med 37: 97–108.

2 Subramanian S, Stolk WA, Ramaiah KD (2004)The dynamics of *Wuchereria bancrofti* infection: a model-based analysis of longitudinal data from Pondicherry, India. Parasitology 128: 467–482.

3 Coffeng LE, Bakker R, Montresor A, de Vlas SJ (2015) Feasibility of controlling hookworm infection through preventive chemotherapy: a simulation study using the individual-based WORMSIM modelling framework. Parasit Vectors 8: 541.

4 Stolk WA, Walker M, Coffeng LE, Basáñez M-G, de Vlas SJ. Required duration of mass ivermectin treatment for onchocerciasis elimination in Africa: a comparative modelling analysis. *Parasit Vectors* 2015; **8**: 552.

5 Stolk WA, Swaminathan S, Oortmarssen GJ van, Das PK, Habbema JDF (2003) Prospects for elimination of bancroftian filariasis by mass drug treatment in Pondicherry, India: a simulation study. J Infect Dis 188: 1371–1381.

6 Stolk WA, de Vlas SJ, Habbema JDF (2005) Anti-Wolbachia treatment for lymphatic filariasis. Lancet Infect Dis365: 2067–2068.

7 Stolk WA, De Vlas SJ, Habbema JDF. Advances and challenges in predicting the impact of lymphatic filariasis elimination programmes by mathematical modelling. *Filaria J* 2006; **5**: 5.

8 Stolk WA, De Vlas SJ, Borsboom GJ, Habbema JDF (2008) LYMFASIM, a simulation model for predicting the impact of lymphatic filariasis control: quantification for African villages. Parasitology 135: 1583–1598.

9 Stolk WA, ten Bosch QA, de Vlas SJ, Fischer PU, Weil GJ (2013) Modeling the impact and costs of semiannual mass drug administration for accelerated elimination of lymphatic filariasis. PLoS Negl Trop Dis **7**: e1984.

10 Stolk WA, Stone C, de Vlas SJ (2015) Modelling lymphatic filariasis transmission and control: modelling frameworks, lessons learned and future directions. Adv Parasito*l* 87: 249–291.

11 Jambulingam P, Subramanian S, de Vlas SJ, Vinubala C, Stolk WA (2016) Mathematical modelling of lymphatic filariasis elimination programmes in India: required duration of mass drug administration and post-treatment level of infection indicators. Parasit Vectors 9: 501.

12 Irvine MA, Stolk WA, Smith ME (2017) Effectiveness of a triple-drug regimen for global elimination of lymphatic filariasis: a modelling study. Lancet Infect Dis 17: 451–458.

13 Smith ME, Singh BK, Irvine MA (2017) Predicting lymphatic filariasis transmission and elimination dynamics using a multi-model ensemble framework. Epidemics 18: 16–28.

14 World Health Organization (1992) Lymphatic filariasis: the disease and its control. Fifth report of the WHO Expert Committee on Filariasis. World Health Organ Tech Rep Se*r.* 821: 1–71.

15 Plaisier AP, Cao WC, Van Oortmarssen GJ, Habbema JDF. Efficacy of ivermectin in the treatment of *Wuchereria bancrofti* infection: a model-based analysis of trial results. Parasitology 119: 385–394.

16 Subramanian S, Krishnamoorthy K, Ramaiah KD, Habbema JDF, Das PK, Plaisier AP (1998) The relationship between microfilarial load in the human host and uptake and development of *Wuchereria bancrofti* microfilariae by *Culex quinquefasciatus*: a study under natural conditions. Parasitology 116: 243–255.

TRANSFIL model description and methods

**Description of the mathematical model**

The mathematical model of lymphatic filariasis (LF) transmission TRANSFIL is a stochastic individual-based model of LF infection in human populations. A full model description is given in Irvine *et al.*1 and more recently in Michael *et al.*2, so here we provide a brief summary of the model development. TRANSFIL is a stochastic individual-based model, simulating worm burden, microfilaraemia and other demographic parameters relating to age and risk of exposure. Humans are modelled individually, with their own male and female worm burden. The concentration of mf in the peripheral blood is modelled for each individual and increases according to the number of fertile female worms as well as decreasing at constant rate.

The total mf density in the population contributes towards the current density of L3 larvae in the human-biting mosquito population, where the distribution of L3 amongst the human-biting mosquito population is completely homogeneous. An empirically derived relationship is used for the uptake of mf by a mosquito, where both *Culex* and *Anopheles* uptake curves are implemented depending on setting (see Irvine *et al.*1), the relationship with *Anopheles* was used in the main manuscript. The model dynamics are therefore divided into the individual human dynamics, including age and turnover; worm dynamics inside the host; microfilariae dynamics inside the host and larvae dynamics inside the mosquito.

To generate the required range of mf prevalences in individuals above 5 years of age (prevalence range 0 to 60% in the scenarios considered for Ethiopia), we varied three parameters of the model, the vector to host ratio (V/H), the average population bite risk (k) and the importation rate (Imp), using parameter sets from a range of plausible values based on previously analysed data1,2,3. The graphical representation of the values is shown in Figures S5-S7.

For stochastic models it is essential that an importation rate is included, otherwise the equilibrium distribution (steady state) that is used as the starting point of the simulations can potentially converge to the degenerate distribution where no-one is infected. The importation rate does not need to be large, in fact it should not be driving the infection. For this LF study we used a random number drawn from a uniform distribution with minimum 0 and maximum 0.00025 (max 2.5/10000 infections per month). The interventions reduce the prevalence over time, and therefore as year pass, the importation rate decreases in proportion to the reduction in prevalence seen in some pilot simulations. More specifically, we produced 2000 simulations for each scenario with constant importation rate over the years (including the years of MDA). Then, for our main set of simulations, we adjust the importation rate according to how the prevalence changed in our pilot runs after the intervention was applied (for example see Figure S8).


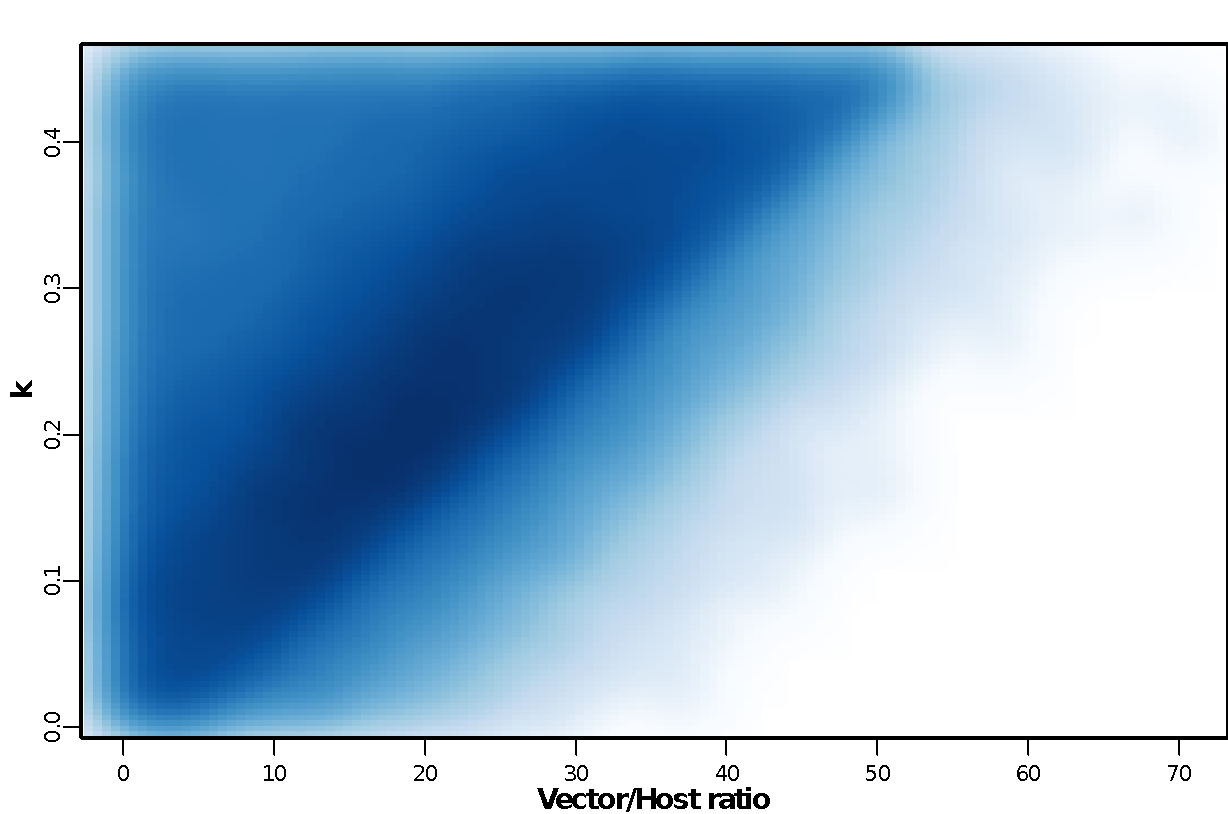


**Figure S4** – Vector to Host ratio against aggregation parameter k. The density plot indicates the parameter space areas from the simulations that were in the 0 to 60% mf prevalence range at baseline. Dark blue areas denote more common parameter values.


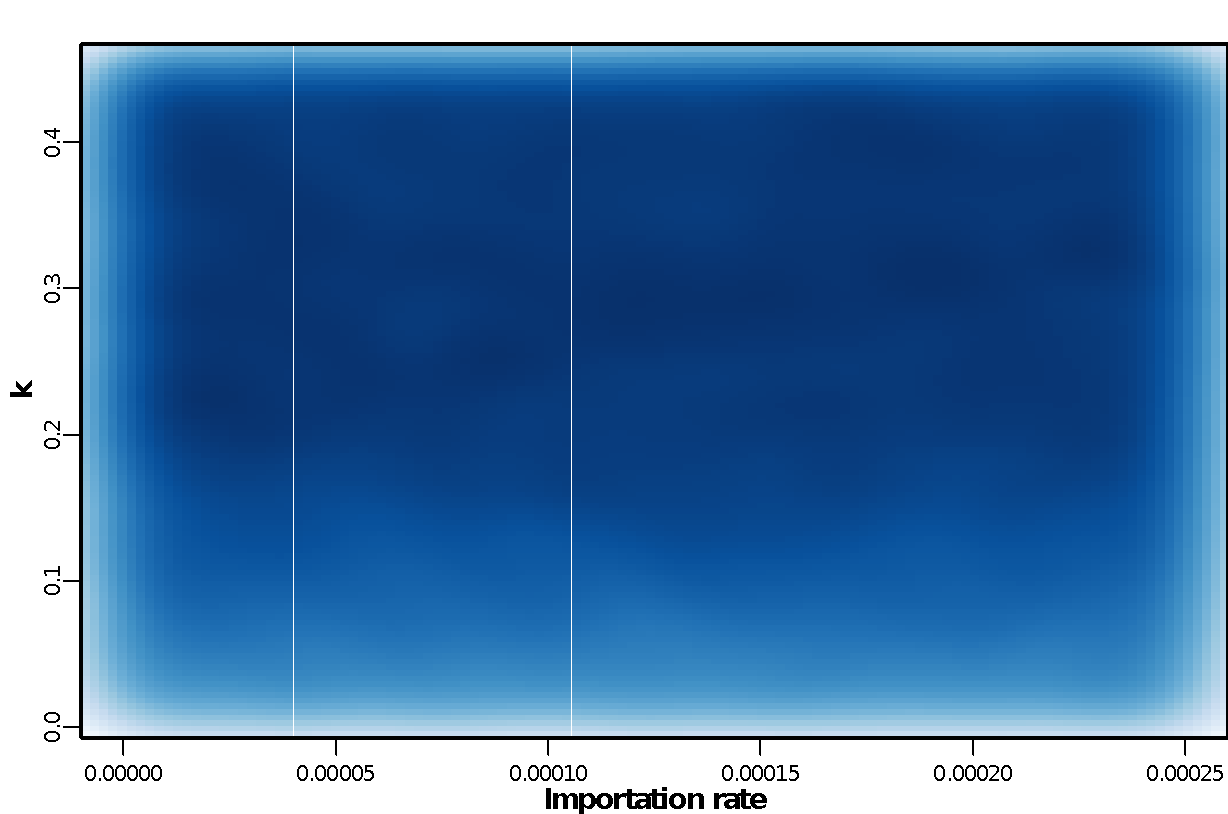


**Figure S5** – Importation rate against aggregation parameter k. The density plot indicates the parameter space areas from the simulations that were in the 0 to 60% mf prevalence range at baseline. Dark blue areas denote more common parameter values.


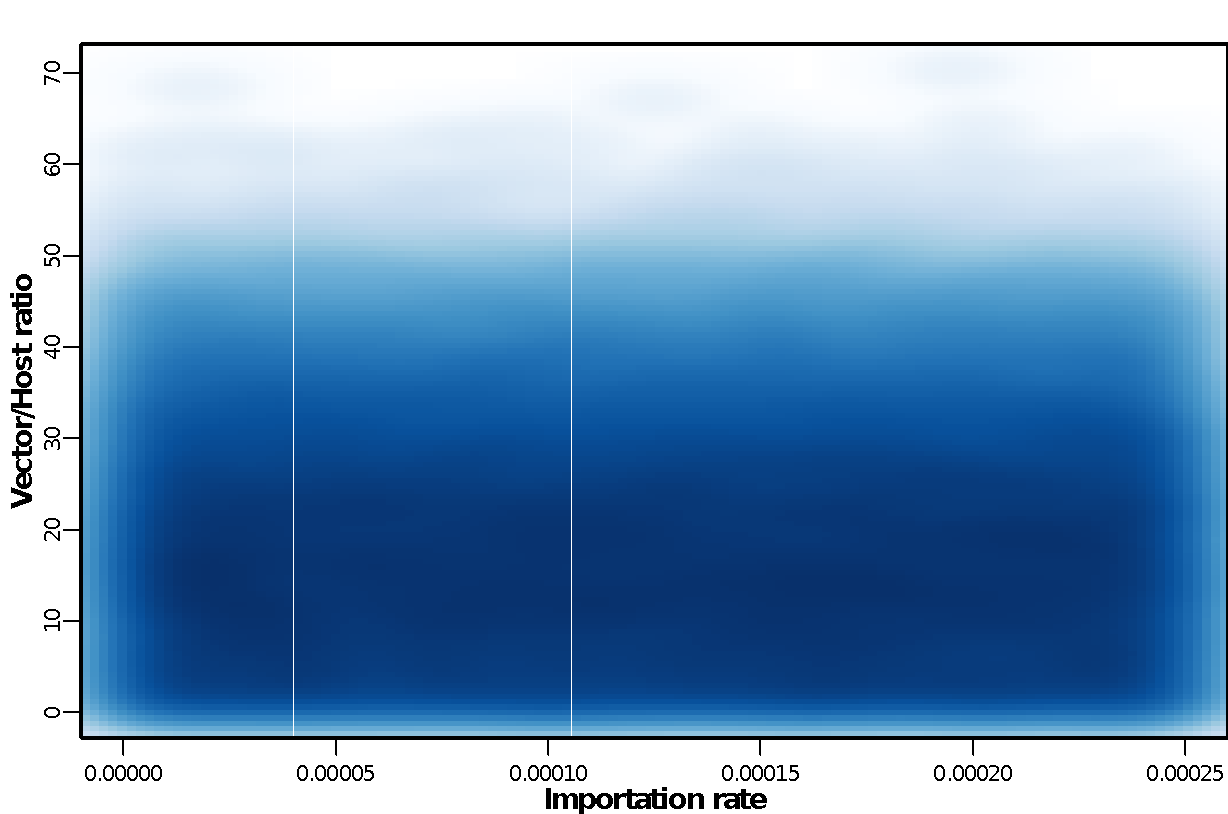


**Figure S6** – Importation rate against Vector to Host ratio. The density plot indicates the parameter space areas from the simulations that were in the 0 to 60% mf prevalence range at baseline. Dark blue areas denote more common parameter values.


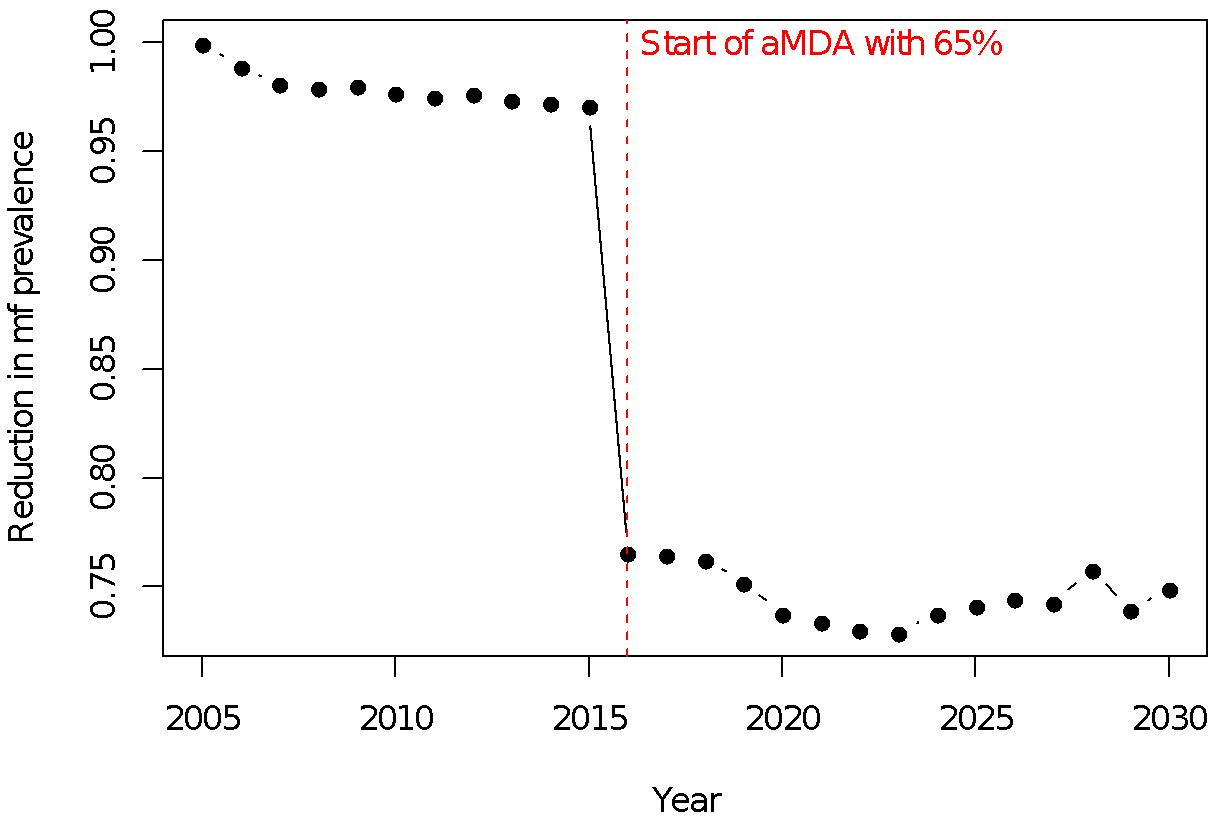


**Figure S7** – Yearly reduction in mf prevalence seen in the pilot simulations. In this scenario (SW_Scen18) the first intervention took place in 2016, annual MDA with 65% coverage, and was applied for 15 rounds.

Compliance between rounds of MDA is modelled based on the paper by Griffin *et al.*14, following the description in Dyson *et al.*15 and previous implementation of the model2. A summary of all model parameters is available in Table S4. Efficacy of MDA and bednets is as with previous implementation of the model16.

**Table S4** – Description the basic LF model parameters.

| **Parameter symbol** | **Definition** | **Value** | **Source** |
| --- | --- | --- | --- |
| λ | Number of bites per mosquito | 10 per month | [4,5] |
| V/H | Ratio of number of vectors to hosts | Varied | Input |
| αmax | Age at which exposure to mosquitoes reaches its maximum level | 20.0 | [6] |
| ψ1 | Proportion of L3 leaving mosquito per bite | 0.414 | [7] |
| ψ2 | Proportion of L3 leaving mosquito that enter host | 0.32 | [8] |
| s2 | Proportion of L3 entering host that develop into adult worms | 0.00275 | [9,10] |
| μ | Death rate of adult worms | 0.0104 per month | [11] |
| δ | Production rate of mf per worm | 0.2 per month | [7] |
| ζ | Death rate of mf | 0.1 per month | [7,12] |
| g | Proportion of mosquitoes which pick up infection when biting an infected host | 0.37 | [13] |
| σ | Death rate of mosquitoes | 5 per month | [8] |
| k | Aggregation parameter of individual exposure to mosquitoes | Varied | Input |
| h(α) | Parameter to adjust rate at which individuals of age α are bitten | Linear from 0 to 10, with maximum of 1 | [9] |
| Imp | Importation rate | Varied | Input |

**References**

1. Irvine MA, Reimer LJ, Njenga SM, Gunawardena S, Kelly-Hope L, Bockarie M, and Hollingsworth TD (2015) Modelling strategies to break transmission of lymphatic filariasis - aggregation, adherence and vector competence greatly alter elimination. Parasites and Vectors 8:547.

2. Michael E, Sharma S, Smith ME, Touloupou P, Giardina F, Prada JM, Stolk WA, Hollingsworth TD, de Vlas SJ (2018) Quantifying the value of surveillance data for improving model predictions of lymphatic filariasis elimination. PLoS Negl Trop Dis 12(10): e0006674.

3. Irvine, M.A., Stolk, W.A., Smith, M.E., Subramanian, S., Singh, B.K., Weil, G.J., Michael, E., Hollingsworth, T.D. (2017) Effectiveness of a triple-drug regimen for global elimination of lymphatic filariasis: a modelling study. Lancet Infectious Diseases 17(4), 451–458.

4. Rajagopalan P (1980) Population dynamics of culex pipiens fatigans, the filariasis vector, in pondicherry: influence of climate and environment. Proc Indian Natl Sci Acad. 6**:** 745–752.

5. Subramanian S, Manoharan A, Ramaiah K, Das P (1994) Rates of acquisition and loss of wuchereria bancrofti infection in culex quinquefasciatus. Am J Trop Med Hyg. 51: 244–249.

6. Subramanian S, Stolk W, Ramaiah K, Plaisier A, Krishnamoorthy K, Van Oortmarssen G (2004). The dynamics of wuchereria bancrofti infection: a model-based analysis of longitudinal data from Pondicherry, India. Parasitology 128: 467–482.

7. Hairston NG, de Meillon B (1968) On the inefficiency of transmission of wuchereria bancrofti from mosquito to human host. Bull World Health Organ. 38: 935.

8. Ho BC, Ewert A (1967) Experimental transmission of filarial larvae in relation to feeding behaviour of the mosquito vectors. Trans R Soc Trop Med Hyg. 61: 663–666.

9. Norman R, Chan MS, Srividya A, Pani S, Ramaiah KD, Vanamail P (2000) EPIFIL: The development of an age-structured model for describing the transmission dynamics and control of lymphatic filariasis. Epidemiol Infect. 124: 529–541.

10. Stolk WA, De Vlas SJ, Borsboom GJ, Habbema J (2008) LYMFASIM, a simulation model for predicting the impact of lymphatic filariasis control: Quantification for African villages. Parasitology 135L: 1583–1598.

11. Evans DB, Gelband H, Vlassoff C (1993) Social and economic factors and the control of lymphatic filariasis: a review. Acta Trop. 53: 1–26.

12. Ottesen E, Ramachandran C (1995) Lymphatic filariasis infection and disease: control strategies. Parasitol Today 11: 129–130.

13. Subramanian S, Krishnamoorthy K, Ramaiah K, Habbema J, Das P, Plaisier A (1998) The relationship between microfilarial load in the human host and uptake and development of wuchereria bancrofti microfilariae by culex quinquefasciatus: a study under natural conditions. Parasitology. 116: 243–255.

14. Griffin JT, Hollingsworth TD, Okell LC, Churcher TS, White M, Hinsley W, Bousema T, Drakeley CJ, Ferguson NM, Basez MG, and Ghani AC. (2010) Reducing Plasmodium falciparum malaria transmission in Africa: a model-based evaluation of intervention strategies. PLOS Medicine. **7**: 1-17.

15. Dyson L, Stolk WA, Farrell SH, and Hollingsworth TD (2017) Measuring and modelling the effects of systematic non-adherence to mass drug administration. Epidemics 18: 56-66.

16. Stolk WA, Prada JM, Smith ME, Kontoroupis P, de Vos AS, Touloupou P, Irvine MA, Brown P, Subramanian S, Kloek M, Michael E, Hollingsworth TD, de Vlas SJ. Are Alternative Strategies Required to Accelerate the Global Elimination of Lymphatic Filariasis? Insights From Mathematical Models. Clin Infect Dis. 2018 Jun 1;66(suppl_4):S260-S266. doi: 10.1093/cid/ciy003.

Validation of model integration

**Linking geostatistical and transmission models**

To evaluate the performance of the method described in the main text following1, we illustrate in Figure S9 the observed and the estimated distribution of the baseline prevalence (a) and the population (b) at each pixel. The results indicate that the method performs very well, since most of the points lie near the red line x = y. We further assessed the performance of the method by presenting in Figure S10 the histogram of the observed baseline prevalence for a sample pixel (a) and the corresponding weighted histogram of the estimated prevalence (b). The plots are essentially identical as expected.

| 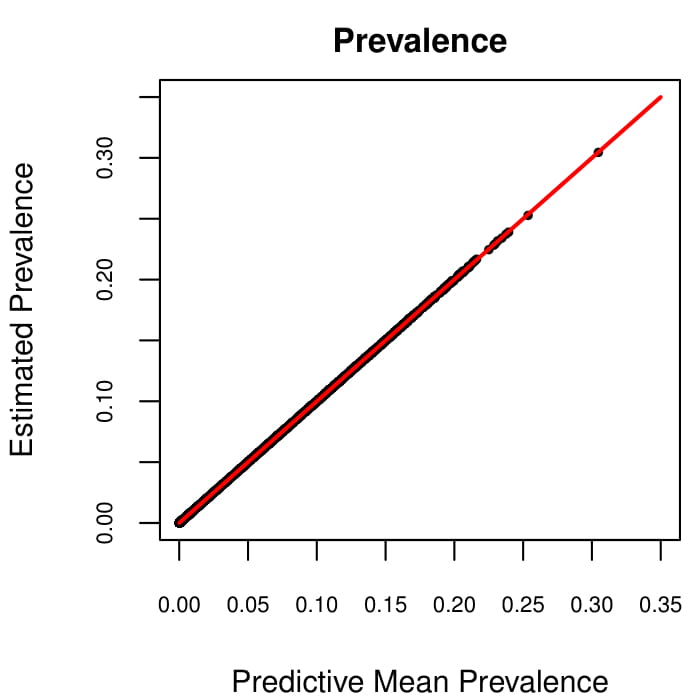   1. Comparison of the estimated baseline prevalence per pixel with the observed value. | 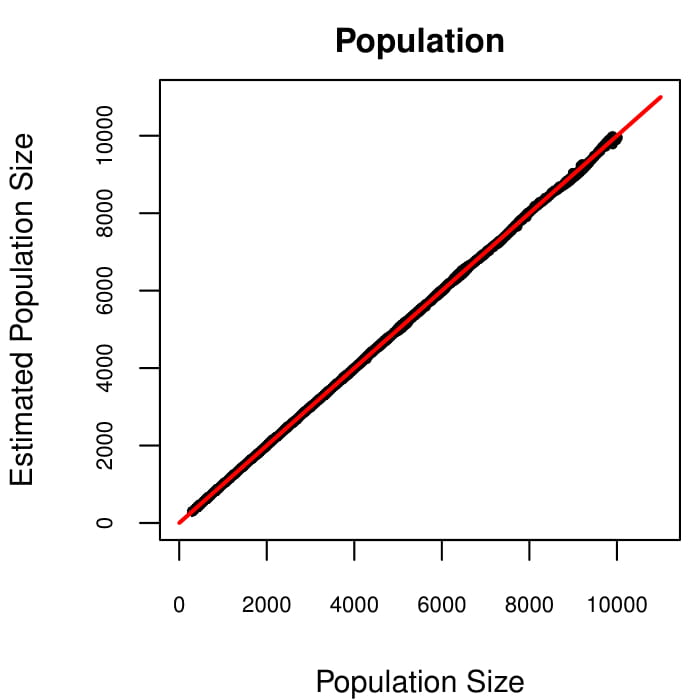   1. Comparison of the estimated population size per pixel with the observed value. |
| --- | --- |

**Figure S8** - Comparison between estimated and predicted prevalence and population size.

| 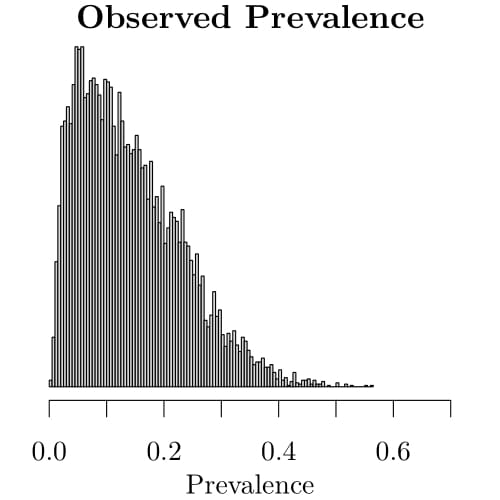   1. Histogram of the observed baseline prevalence for a sampled pixel. | 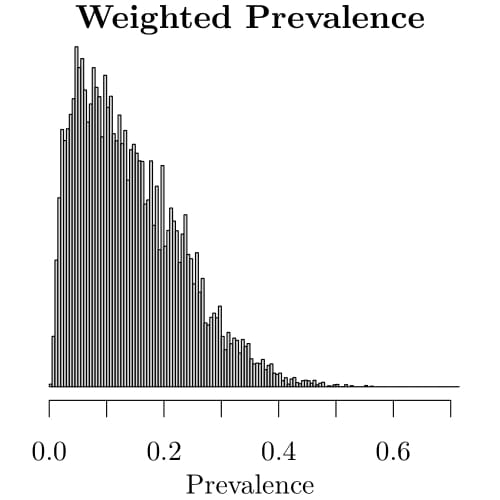   1. Weighted histogram of the estimated baseline prevalence for the sampled pixel. |
| --- | --- |

**Figure S9** - Comparison between observed and weighted (estimated) baseline prevalence for a sample pixel.

**References**

1. Touloupou, P., Retkute, R., Hollingsworth, T. D. & Spencer, S. E. F. Statistical methods for linking geostatistical maps and transmission models: Application to lymphatic filariasis in East Africa. Spat Spatiotemporal Epidemiol 41, 100391, doi:10.1016/j.sste.2020.100391 (2022).

Historical intervention scenarios


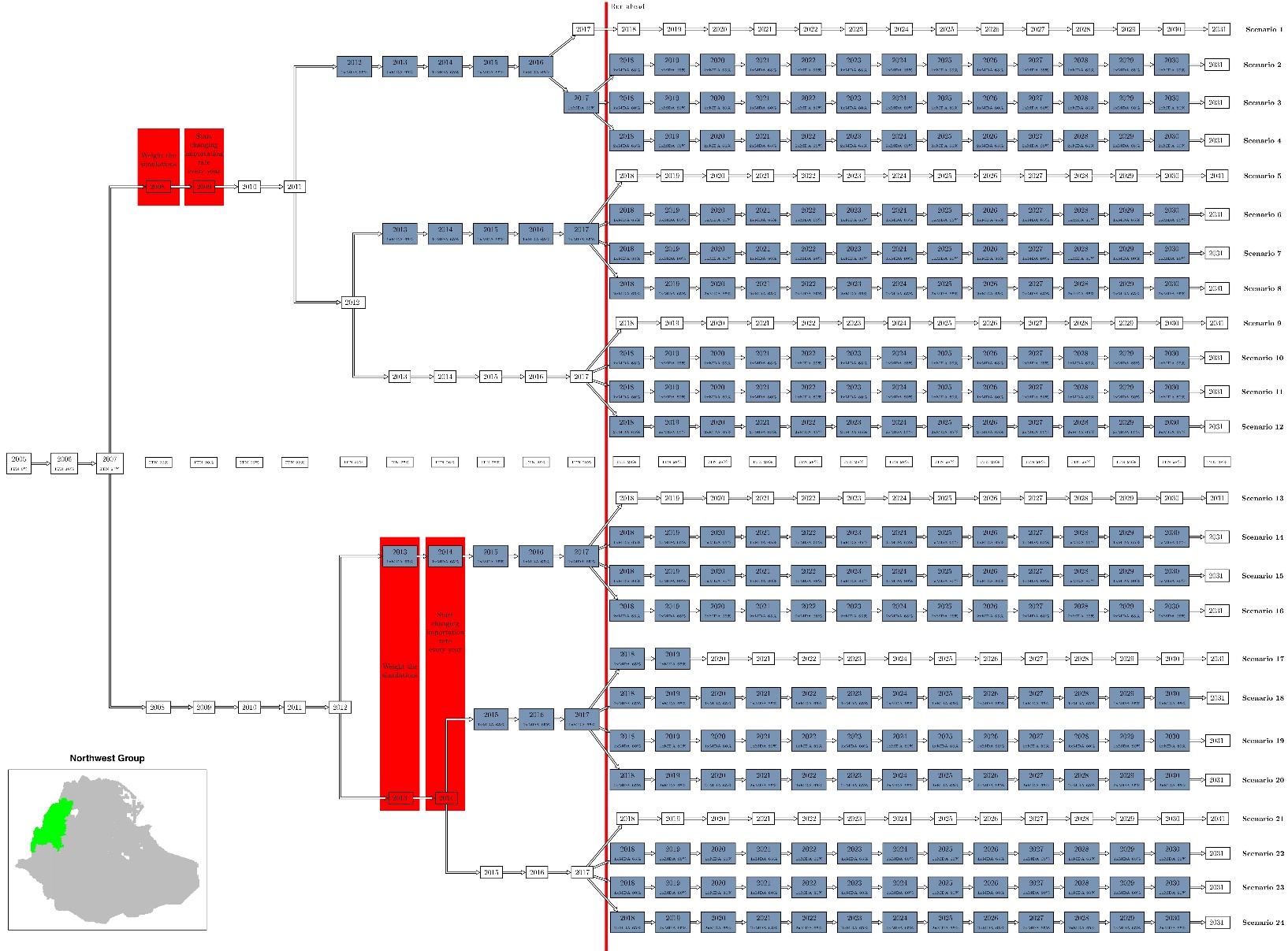


**Figure S10** - Scenarios for the pixels in the Northwest group, which includes the regions coloured in green, pixels can be further divided into 7 groups resulting in 24 scenarios.


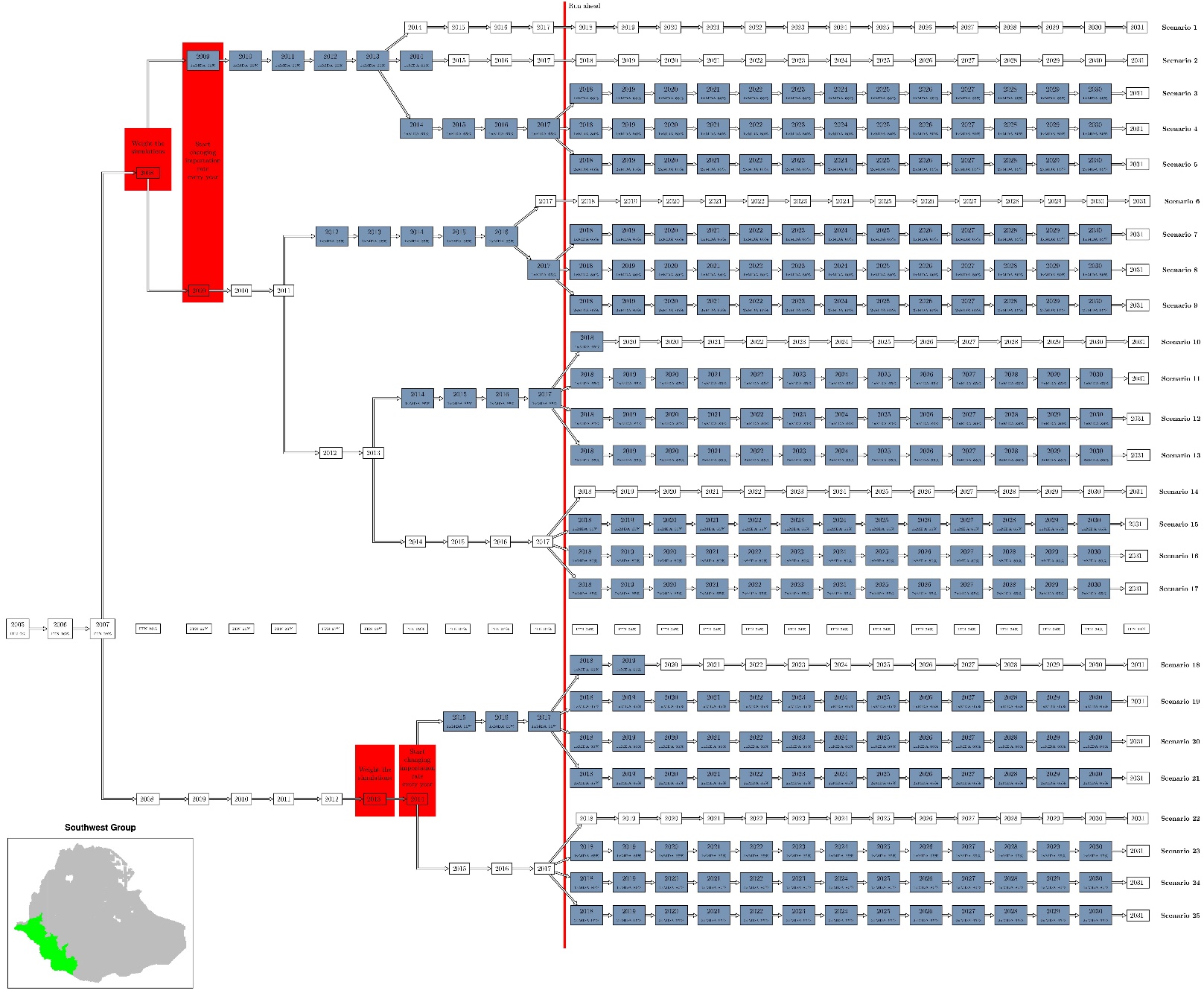


**Figure S11** - Scenarios for the pixels in the Southwest group, which includes the regions coloured in green, pixels can be further divided into 9 groups resulting in 25 scenarios.


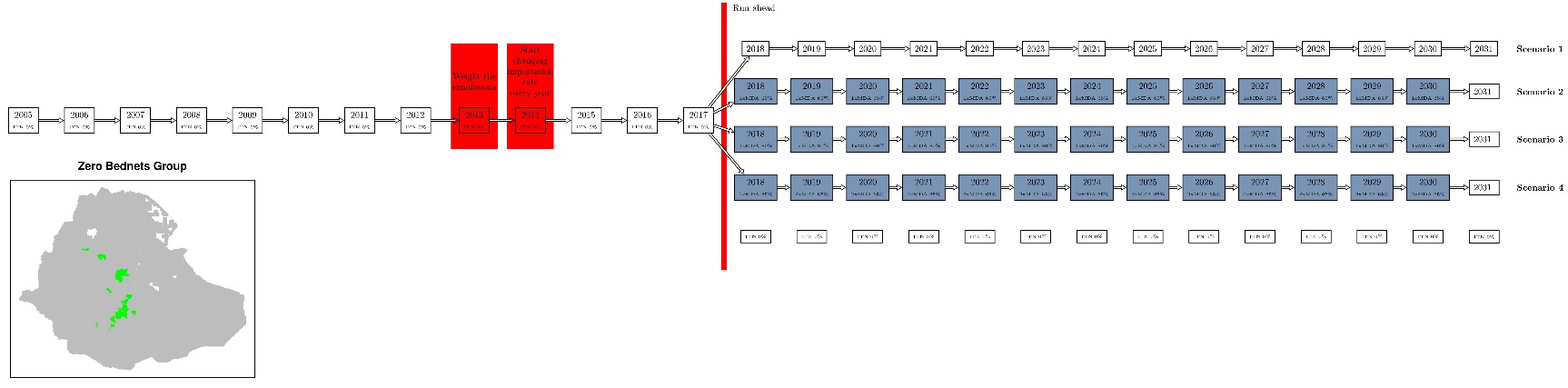


**Figure S12** - Scenarios for the pixels in the zero bed-net coverage group, which includes the regions coloured in green, we considered 4 scenarios.


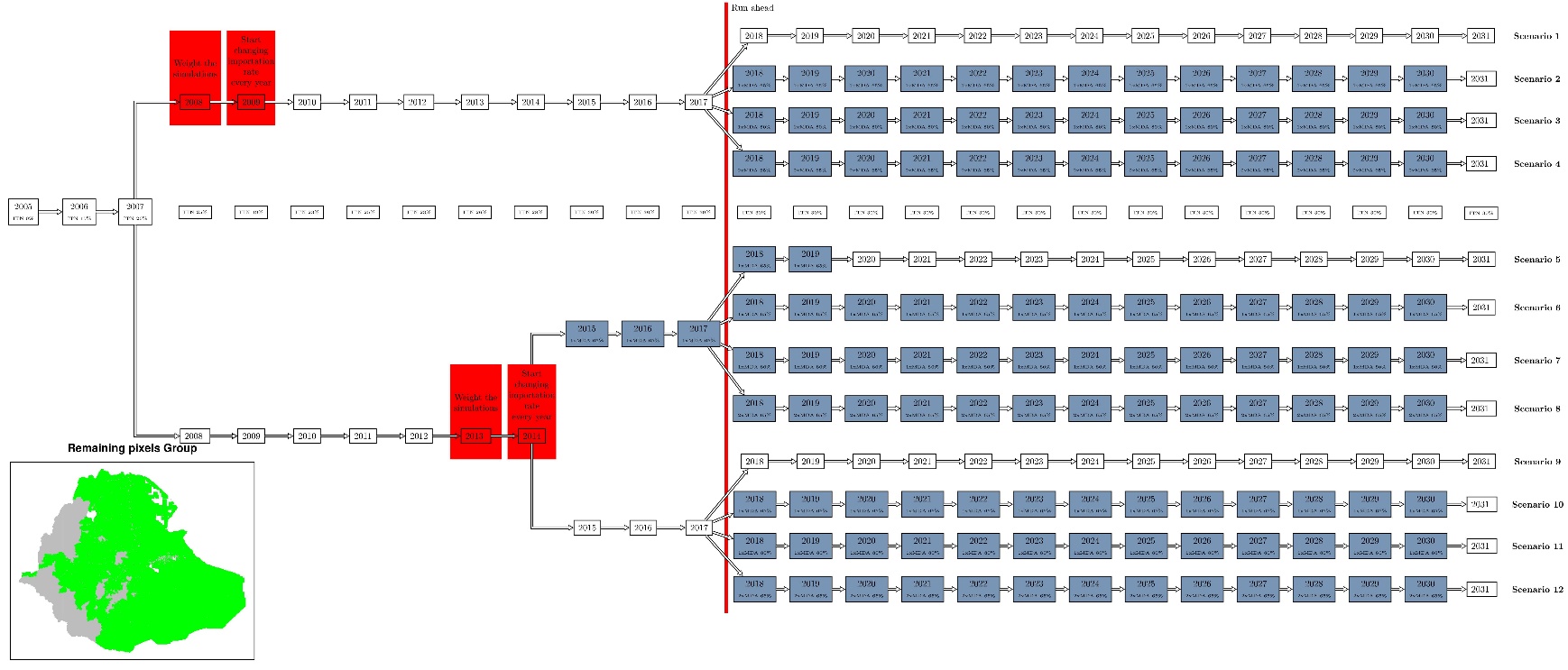


**Figure S13** - Scenarios for the pixels in the remaining group, which includes the regions coloured in green, pixels can be further divided into 3 groups resulting in 12 scenarios.

Pixel-level 2020 projections


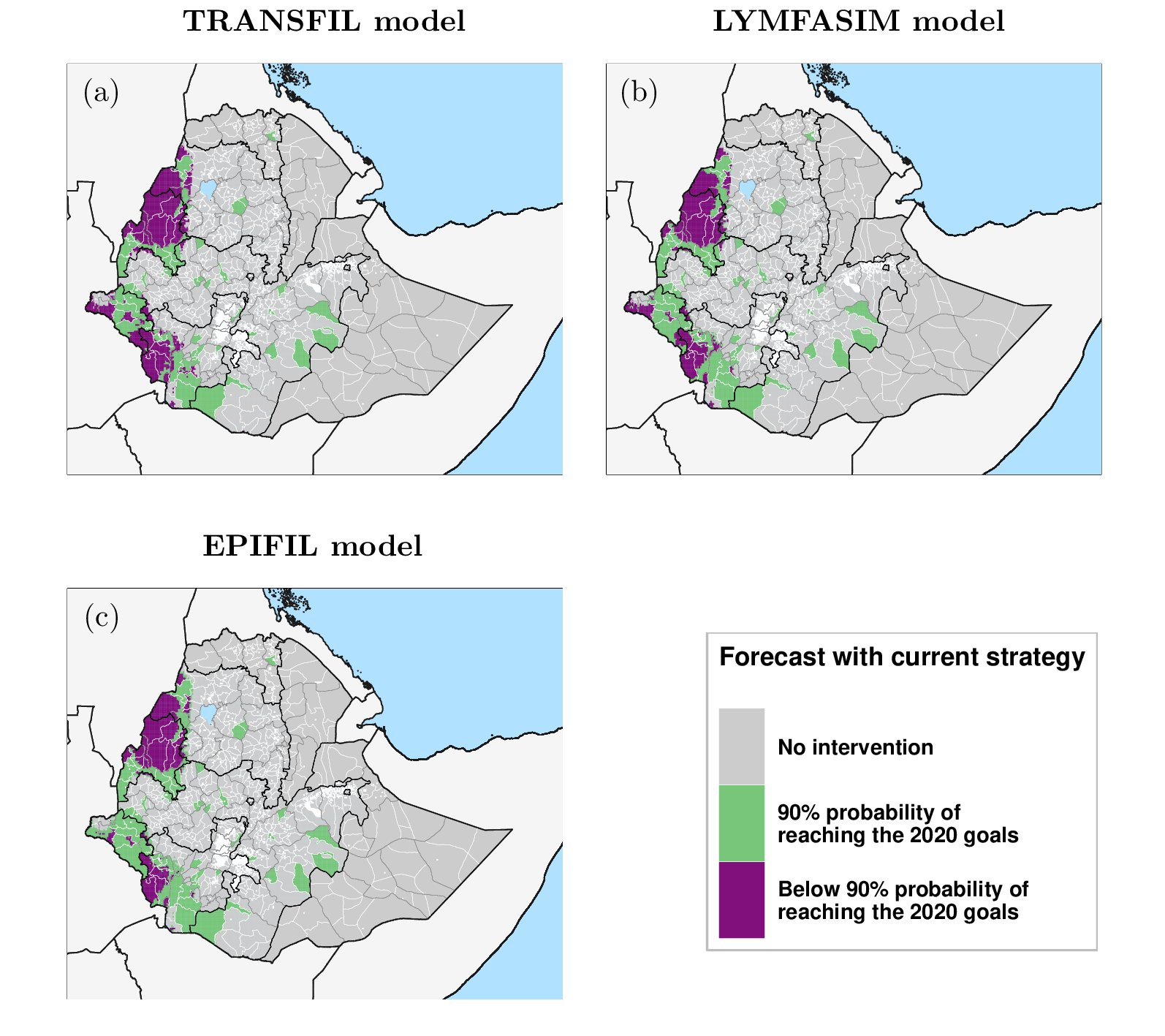


**Figure S14** – Pixel-level forecast for reaching 1% mf prevalence by 2020 with 90% probability, under the “current intervention” strategy with TRANSFIL (a), LYMFASIM (b) and EPIFIL (c). Grey areas are considered non-endemic. Pixels in green have a probability above 90% of reaching EPHP (1% mf prevalence), while pixels in purple have a probability below 90% of reaching it.

Lymphatic filariasis elimination status in 2020


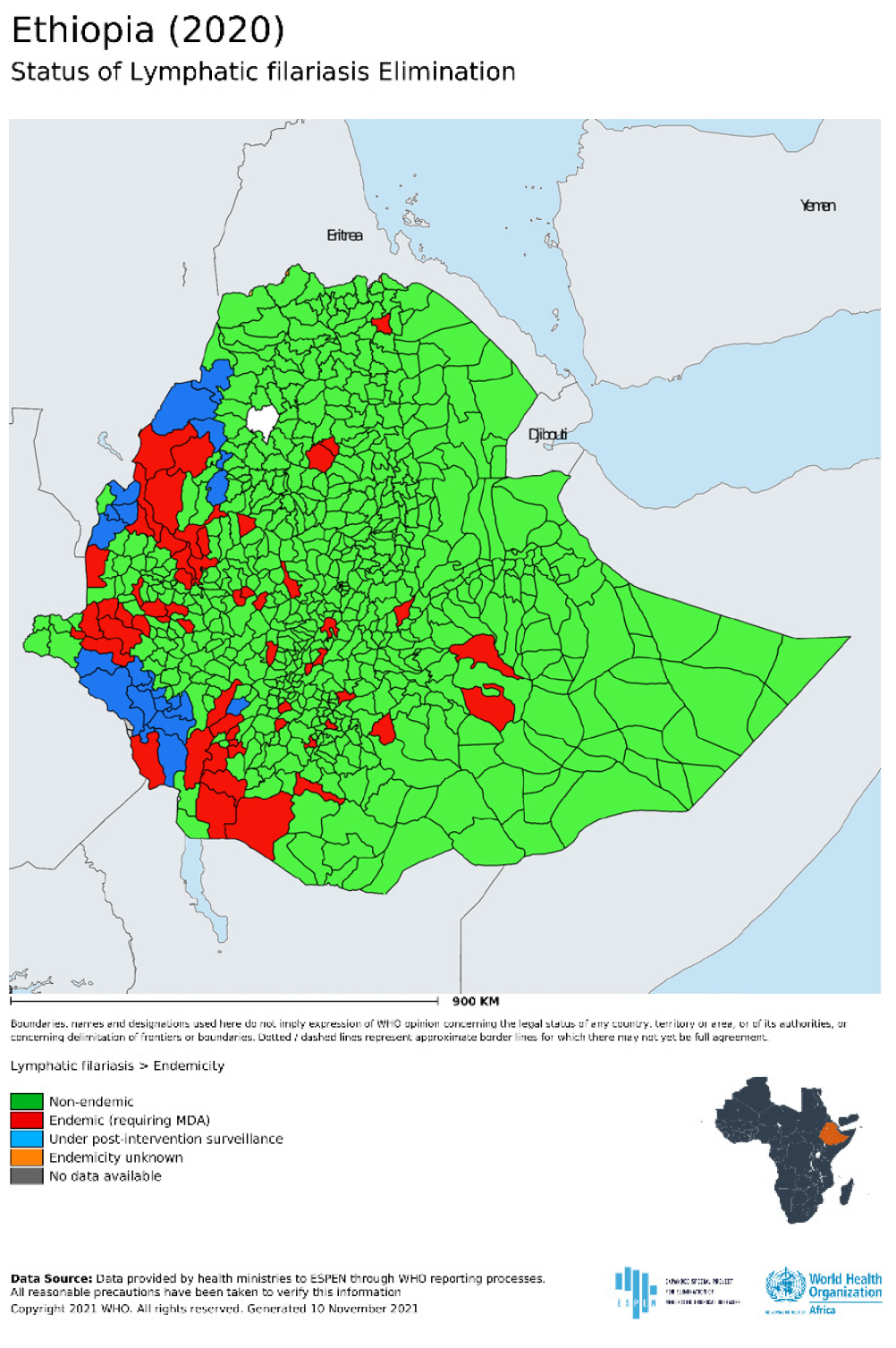


**Figure S15** – Woreda classification in Ethiopia regarding lymphatic filariasis elimination status in 2020, reproduced from “The Third National Neglected Tropical Diseases Strategic Plan 2021-2025”

(Ministry of Health – Ethiopia).

1. Children under 5 are assumed to be exempted from treatment [↑](#footnote-ref-1)
2. Assuming complete absence of mf production during this period and immediate resumption of mf production thereafter [↑](#footnote-ref-2)
